# Investigations of the Molecular Nature of Low-Molecular-Mass Copper(II) Ion Complexes in Human Saliva: Physiological and Toxicological Significance

**DOI:** 10.3390/molecules31152686

**Published:** 2026-08-01

**Authors:** Kayleigh Hunwin, Oliver Megram, Georgina Page, Wyman Chan, Nazmin Juma, Rachel Armitage, Olivia Steel, Katie Jane Hewitt, Martin Grootveld

**Affiliations:** 1 Leicester School of Pharmacy, De Montfort University, Leicester LE1 9BH, UK; p2421485@my365.dmu.ac.uk (K.H.); oliver.megram@dmu.ac.uk (O.M.); georgina.page@dmu.ac.uk (G.P.); smilestudiouk@aol.com (W.C.); njuma@dmu.ac.uk (N.J.); olivia.steel@dmu.ac.uk (O.S.); p2585316@my365.dmu.ac.uk (K.J.H.); 2 School of Heritage and Culture, University of Leicester, University Road, Leicester LE1 7RH, UK; ra486@leicester.ac.uk

**Keywords:** copper(II) ion speciation, human saliva, ^1^H NMR spectroscopy, FTIR-ATR, metal ion–biomolecule interactions, dietary copper, metallic taste, bioavailability, toxicology

## Abstract

Copper is an essential trace element that plays critical roles in enzymatic processes and cellular metabolism. However, excessive exposure to its ions can lead to toxicity through oxidative stress mechanisms. Therefore, the chemical ‘speciation’ of copper(II) ions (Cu(II)) in biological fluids such as saliva is of critical importance. However, currently this phenomenon remains poorly understood, despite its importance for our understanding of bioavailability, toxicity, and sensory perception. Therefore, this study employed ^1^H nuclear magnetic resonance (NMR) spectroscopy, Fourier-transform infrared attenuated total reflectance (FTIR-ATR) spectroscopy, and scanning electron microscopy (SEM) to investigate the molecular nature and complexation characteristics of low-molecular-mass Cu(II) complexes formed in human whole mouth salivary supernatant (WMSS) samples. Findings obtained revealed that Cu(II) ions form distinct complexes with salivary biomolecules, particularly amino acids such as histidine and glutamine, aromatic metabolites, proteins, and further small organic biomolecules, e.g., carboxylic acid anions. These results provide new insights into copper speciation in the oral cavity, with implications for dietary Cu(II) ion availability, metallic taste perception, and potential toxicological risks associated with elevated oral exposure levels.

## 1. Introduction

Copper (Cu) ions are essential micronutrients required for numerous physiological processes in humans, functioning primarily as a redox-active catalytic cofactor in enzymes that cycle between the Cu(I) and Cu(II) oxidation states. Copper-dependent enzymes, such as cytochrome *c* oxidase, Cu/Zn superoxide dismutase, ceruloplasmin, and lysyl oxidase, play crucial roles in energy metabolism, antioxidant defence, iron homeostasis, and connective tissue formation [1,2,3,4,5,6]. The recommended dietary allowance for copper in adults is 900 μg/day, with tolerable upper intake levels established at 10 mg/day [2]. Despite its essentiality, copper exhibits a narrow margin between physiological requirement and toxicity, with both deficient and excess levels associated with significant adverse health outcomes. Systemic copper homeostasis is maintained through regulated intestinal absorption, hepatic storage and redistribution via ceruloplasmin, and biliary excretion [1,3,4,5]. Wilson’s disease, an autosomal recessive disorder caused by mutations in the ATP7B gene, results in pathological copper accumulation in the liver, brain, and other tissues ascribable to impaired biliary copper excretion [7].

Conversely, Menkes disease, caused by ATP7A gene mutations, leads to copper deficiency and severe neurological impairment [8], and copper toxicity can arise from environmental or dietary exposure, including sources involving contaminated drinking water, acidic foods prepared in uncoated copper cookware, or excessive supplementation [9]. The gastrointestinal tract is particularly sensitive to copper exposure, with acute effects observed at doses exceeding approximately 5 mg Cu per day [10]. Severe cases of copper poisoning can progress to haemolytic anaemia, hepatotoxicity, renal failure, and multi-organ dysfunction [11].

Human saliva is a complex biofluid secreted by major and minor salivary glands, mirrors systemic trace element concentrations to some degree, and serves as a non-invasive indicator of nutritional and environmental metal/metal ion exposure [12,13]. Indeed, the oral cavity represents the first site of copper exposure from dietary sources and drinking water [14]. Normal salivary copper concentrations typically range from 4.87 ± 1.10 µmol/L [15], although these levels can fluctuate quite markedly based on dietary intake, pregnancy, drinking water quality, systemic diseases, and the presence of copper-containing dental materials such as orthodontic appliances. Occupations involving metal exposure, such as welding, have been shown to correlate with elevated salivary copper levels, suggesting that external exposure pathways can influence salivary Cu(II) content and potentially reflect systemic perturbations [14]. However, the substantial accumulation of copper has not been observed in patients with Wilson’s disease [16], despite systemic copper overload, a process suggesting distinct regulatory mechanisms for salivary copper homeostasis. The biochemical speciation of copper ions in saliva (that is, the specific molecular forms of it, and complexes in which copper ions exist) critically influences its bioavailability, toxicity, and sensory properties, as might be anticipated.

Previously conducted research studies have shown that soluble copper species are primarily responsible for metallic taste perception, whilst insoluble or protein-bound copper may contribute to astringency through interactions with salivary proteins [17]. At physiological salivary pH (approximately 6.8–7.2), a significant proportion of added copper forms insoluble precipitates with and/or binds to salivary macromolecules, thereby reducing the concentration of free, “bioavailable” copper ions [18].

Saliva contains a very wide range of natural biomolecules, including proteins, peptides, amino acids, urea, glucose, lipids, and other metabolites, along with inorganic ions (including those of sodium, potassium, calcium, chloride, bicarbonate, and phosphate) [12,19,20,21,22]. Major salivary amino acids, organic acid anions and proteins exhibit significant binding affinities for metal ions such as Co(II) and Cu(II), altering their speciation status, solubility, and biological availability in the oral cavity [23].

Amongst salivary proteins, those with molecular masses of approximately 29 kDa and 33 kDa have been identified as forming insoluble complexes with Cu(II) ions at elevated concentrations. Low-molecular-mass mucin (MG2), α-amylase, basic proline-rich proteins, and a 45 kDa protein also exhibit Cu(II)-binding capacity [18]. Histatin-5, a salivary antimicrobial peptide present at 15–50 µM concentrations, has been identified as a particularly important Cu(II)-binding ligand, forming high-affinity complexes through its ATCUN-binding motif [24].

Despite these advances, the detailed molecular characterisation of copper speciation in human saliva has remained incomplete. Nuclear magnetic resonance (NMR) spectroscopy (predominantly ^1^H NMR analysis) offers unique advantages for investigating metal ion speciation in complex biological matrices. Unlike techniques that require separation or digestion of samples, NMR analysis provides direct, non-destructive molecular-level information about metal–ligand interactions, chemical environments, and dynamic exchange processes in the solution [25]. For paramagnetic metal ions such as Cu(II), the unpaired electrons can induce significant effects on the NMR spectra of coordinated ligands, including line broadening, chemical shift perturbations, and paramagnetic relaxation enhancement (PRE). These effects can be exploited to identify copper(II)-binding sites, and also characterise the coordination geometry of copper complexes.

^1^H NMR spectroscopy has proven particularly valuable for investigating the speciation of metal ions in biofluids [26,27,28]. Recent work from our group has demonstrated the power of ^1^H NMR analysis to elucidate the coordination chemistry of nickel(II) and copper(II) ions in RPMI 1640 cell culture medium, revealing complex formation with amino acids and organic acid anions [29]. These findings have highlighted how transition metal ion coordination can alter their physicochemical properties such as redox potentials, membrane permeability, and their capacities for reactive oxygen species (ROS) generation. Similar approaches have been applied to study metal complexes of pharmaceutical relevance, including platinum(II), gold(I), ruthenium(II) and (III), and bismuth(III) compounds, providing mechanistic insights into their interactions with plasma proteins and intracellular targets [30].

Therefore, the objectives of this study were three-fold: (1) to characterise the ^1^H NMR spectroscopic profiles of human whole mouth salivary supernatant (WMSS) samples, and identify the major endogenous metabolites and biomolecules present; (2) to investigate the effects of Cu(II) addition on salivary ^1^H NMR spectra, with particular attention to concentration-dependent changes in resonance intensities, line-widths, and chemical shift values that reflect copper(II) ion–biomolecule interactions; and (3) to employ complementary FTIR-ATR spectroscopy and SEM imaging to validate and extend these NMR findings, particularly those regarding the molecular nature of precipitate formation and associated structural modifications. By elucidating the molecular nature of Cu(II) complexes in human saliva, this research aims to enhance our understanding of copper bioavailability, the physicochemical basis of metallic taste perception, and potential toxicological mechanisms relevant to dietary copper ion exposure and oral copper ion release from dental materials.

## 2. Results

### 2.1. ^1^H NMR Analysis of WMSS Samples

**Figure 1:**
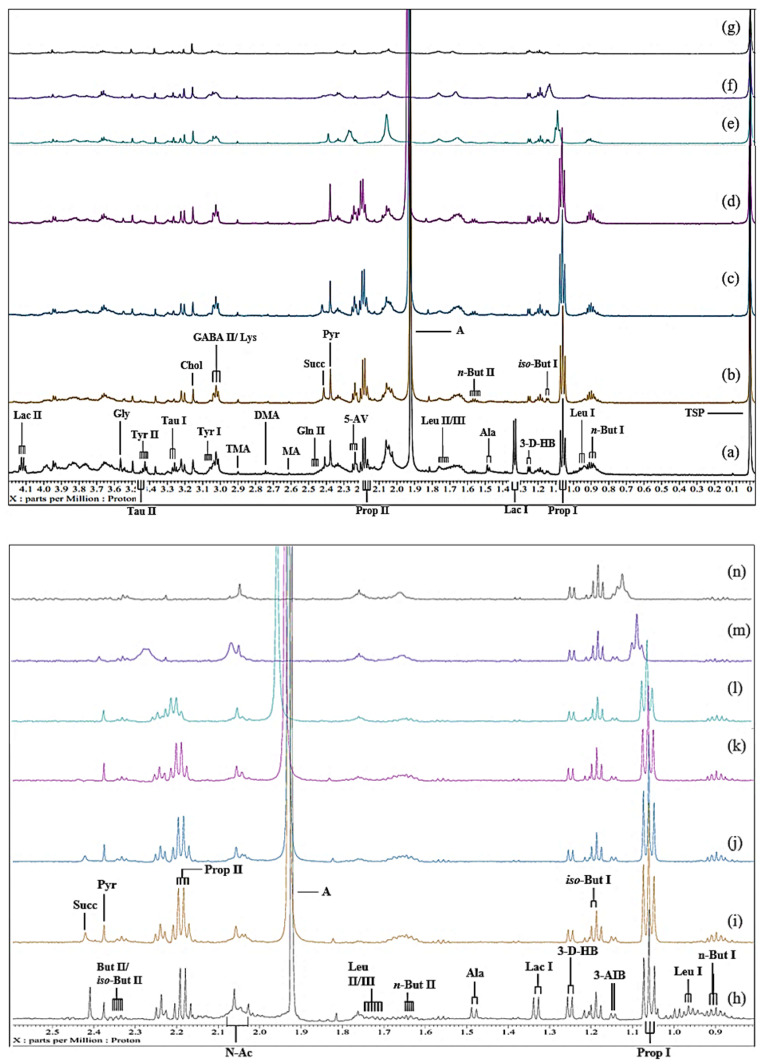
Expanded 0.00–4.20 ppm region of the 600.17 MHz ^1^H NMR spectra of (**a**) a control human whole mouth salivary supernatant (WMSS) sample, and the same sample following equilibration with Cu(II) at added concentrations of (**b**) 7.1 µmol/L, (**c**) 14, (**d**) 28, (**e**) 42, (**f**) 54 and (**g**) 67 µmol/L (top profile), with (**h**–**n**) showing the expanded 0.80–2.60 ppm regions of the spectra displayed in (**a**), (**b**), (**c**), (**d**), (**e**), (**f**) and (**g**), respectively (bottom profile). Typical spectra are shown. Abbreviations: A, acetate-CH_3_; 3-AIB, 3-aminoisobutyrate-CH_3_; Ala, alanine-CH_3_; 5-AV, 5-aminovalerate-δ-CH_2_; 3-D-HB, 3-D-hydroxybutyrate-CH_3_; *iso*-But I, *iso*-butyrate-CH_3_s; n-But I and II, n-butyrate-CH_3_ and β-CH_2_ protons, respectively; GABA-II, γ-aminobutyrate γ-CH_2_; Gln II, glutamine-γ-CH_2_; Gly, glycine-CH_2_; Lac I and II, lactate-CH_3_ and -CH protons, respectively; Leu-I, II and III, leucine-CH_3_s, β-CH and γ-CH_2_, respectively; Lys, lysine ε-CH_2_ group; MA, DMA and TMA, -N(CH_3_)_n_ group, resonances of methylamine (n = 1), dimethylamine (n = 2) and trimethylamine (n = 3), respectively; N-Ac, region for acetamido methyl groups (i.e., -NHCOCH_3_) of N-acetylsugars (the sharper signals are conceivably ascribable to free sugars, or to low-molecular-mass oligosaccharide fragments containing these sugars, the broader one(s) to those present in the molecularly mobile portions of N-acetylated glycoproteins (GlycA signal)); Prop-I and II, propionate-CH_3_ and -CH_2_, respectively; Pyr, pyruvate-CH_3_; Succ, succinate-CH_2_; Tau I and II, taurine-CH_2_-NH_2_ and ^−^O_3_SCH_2_- proton resonances respectively; Tyr I and II, tyrosine-β-CH_2_ and α-CH protons, respectively; TSP, trimethylsilyl-[2,2,3,3-^2^H_4_] propionate-Si(CH_3_)_3_.

**Figure 2:**
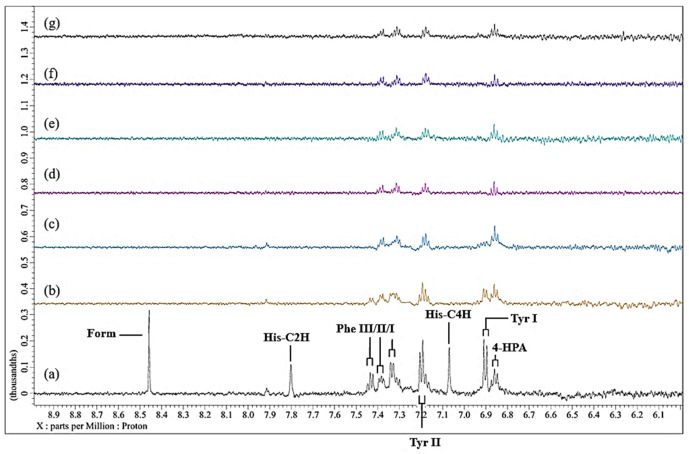
Expanded 5.00–9.00 ppm regions of 600.17 MHz ^1^H NMR spectra of (**a**) a control human WMSS specimen, and the same sample following the addition of (**b**) 7.1, (**c**) 14, (**d**) 28, (**e**) 42, (**f**) 54 and (**g**) 67 µmol/L of Cu(II), showing the complete Cu(II)-mediated removal of the histidine imidazole ring proton resonances. Typical spectra are shown. Abbreviations: Form, formate-H; His-C2H and -C4H, histidine imidazole ring-C2H and -C4H proton resonances, respectively; 4-HPA, 4-hydroxyphenylacetate-H3/H5 aromatic ring protons; Phe I, II and III, phenylalanine-H2/H6, H4 and H3/H5 aromatic ring protons, respectively; Tyr I and II, tyrosine-H3,H5 and H2,H6 aromatic ring protons, respectively.

Figure 1a and Figure 2a show the upfield (0.00–4.28 ppm) and downfield (5.50–8.98 ppm) regions, respectively, of a representative WMSS ^1^H NMR spectrum. Signals corresponding to short-chain organic acid anions (acetate, n-butyrate, propionate, lactate, pyruvate, succinate, and formate) were observed, alongside resonances from amino acids (e.g., glycine, alanine, glutamine, histidine, phenylalanine, and tyrosine) and carbohydrates, including N-acetyl sugar moieties associated with what is known as the GlycA signal from mobile acute-phase glycoprotein side-chains [12,21]. Broad signals observed in the aromatic regions (≈6.90, 7.12, 7.25, and 7.92 ppm) are attributable to aromatic ring protons of tyrosine, phenylalanine, and/or tryptophan residues present in salivary macromolecules, presumably proteins. Indeed, such biomacromolecules possess shorter *T*_2_ values, reflecting their enhanced line-widths and diminished molecular mobilities.

### 2.2. Spectral Titrations of WMSS Samples with Increasing Added Concentrations of Cu(II)_(aq.)_

After acquisition, all ^1^H NMR profiles of WMSS samples underwent routine visual inspection in which biomolecule resonances therein demonstrated concentration-dependent responses to increasing sequential levels of added paramagnetic Cu(II)_(aq.)_, which were observable as changes in the line-widths, intensities and/or chemical shift values of their resonances.

Figure 1 and Figure 2 show the high- and low-field regions of a spectral titration involving the stepwise addition of final Cu(II) concentrations of 7.1–67.0 µmol/L (as outlined above) to a typical WMSS sample. In particular, clearly visible line-broadening effects were observable for the salivary lactate (δ = 1.33 (*d*) and 4.13 (*q*) ppm), alanine (δ = 1.48 (*d*) ppm), taurine (δ = 3.25 (*t*) and 3.42 (*t*) ppm), glycine (δ = 3.58 (*s*) ppm), tyrosine (δ = 6.88 (*d*) and 7.23 (*d*) ppm), and formate (δ = 8.46 (*s*) ppm) signals at the lowest added concentration of Cu(II), alongside notable broadening of the N-acetyl sugar acetamido-CH_3_ group series of resonances (δ = 1.82 (*dd*) and 2.06 (*s*) ppm). Hence, the importance of these salivary biomolecules to complex this metal ion is clearly highlighted in this dataset.

Furthermore, significant broadenings of resonances attributable to succinate (δ = 2.41 (*s*) ppm) and phenylalanine (δ = 7.32 (*m*), 7.37 (*m*) and 7.43 (*m*) ppm) occur when the added level of Cu(II) is increased to 14 µmol/L. Rises in this metal ion’s concentration to levels of 28 and 41 µmol/L, however, also gave rise to its broadenings of the ^1^H NMRs of methylamine (δ = 2.51 (*s*) ppm), dimethylamine (δ = 2.76 (*s*) ppm) and trimethylamine (δ = 2.91 (*s*) ppm), with propionate (δ = 1.05 (*t*) and 2.17 (*q*) ppm) also undergoing significant broadening when added levels of Cu(II) ion attained 54 µmol/L. Overall, at added Cu(II) concentrations of ≥54 µmol/L, there was generalised broadening of most biomolecules across both the high- and low-field regions of spectra acquired.

Spectro-visual examinations unveiled biomolecules with the most Cu(II)-complexing power, and these became virtually indistinguishable in spectra acquired on WMSS samples at the highest levels of added Cu(II) ion (≥28 µmol/L). Also, in many cases, the line-widths of WMSS signals influenced by the lower added concentrations of Cu(II) experienced further broadening as the addition of Cu(II) increased. Evidence for the involvement of the N-acetyl sugar acetamido-CH_3_ group series in the complexation/chelation of this transition metal in human saliva is provided by Cu(II) ion-induced broadenings observed for it at the lowest added concentration. Potentially, these agents may be present as residues in N-acetylated glycoprotein carbohydrate side-chains, or as free and/or low-molecular-mass oligosaccharides (the former visible as the broad salivary GlycA resonance [12,21], the latter as sharper singlets). Metabolites most visually impacted by the addition of Cu(II) have been summarised in Table 1 below, with the concentration at which these changes were first visibly observed being defined alongside.

The paramagnetic effect of Cu(II) on the ^1^H NMR spectra of its complexes is realised through the paramagnetic relaxation enhancement (PRE), and this is critically dependent on the distance between the Cu(II) centre and adjacent protons. Indeed, the relaxation enhancement’s magnitude is directly proportional to 1/r^−6^, where r represents the Cu(II)–H distance [31,32]. Hence, as this distance gets greater, the paramagnetic effect is reduced quite substantially.

Overall, since the magnetic moment of the unpaired electron has a very high magnitude, protons within 0.90 Å encounter an extreme PRE, and this frequently broadens signals into the baseline. However, protons which are within the useful 0.90 and 1.5–2.0 Å range generally experience significant line-broadening, which permits their measurement, along with the computation of long-range distance confines.

Also notable is that the line-width at half-height (Δ*v*_1/2_) of a single spectral resonance is proportional to *T*_2_, i.e., Δ*v*_1/2_ = 1/π*T*_2_. Moreover, added Cu(II) ions are very effective at reducing *T*_1_ relaxation times in NMR in view of their powerful paramagnetic properties and PRE parameters. This effect significantly diminishes acquisition times in solution-state NMR experiments. With regard to interdependency, for most standard analytes, physical tumbling ensures that *T*_2_ is lower than or equivalent to *T*_1_, which determines the recovery rate of the magnetisation. Since datasets were normalised using the constant sum-normalisation (CSN) approach, ^1^H NMRs, which are Cu(II)-broadened to intensities of zero or close to zero at their final or near-final titration end-points, also give rise to signal intensity increases in those which are unaffected, or relatively unaffected, by this added metal ion. Nevertheless, datasets which were either not row-wise normalised at all, or median-normalised rather than using the CSN strategy, also resulted in increases in the same resonances as those observed using the latter (Supplementary Materials Figures S1.1 and S1.2). Signals influenced in this manner are discussed in Section 2.3.2.

**Table 1:** Salivary metabolite ^1^H NMRs influenced by the addition of increasing Cu(II) concentrations. The added concentration at which these changes were first observed for these resonances, and their assignments in the WMSS spectra acquired, are indicated. As noted in Section 4.4 below, WMSS resonance assignments were confirmed via the application of supporting databases, e.g., the *Human Metabolome Database* (HMDB) [33], in addition to those available from published scientific literature [12]. Further confirmation of these was made using *ChenomX* software (*ChenomX* NMR Suite, Professional Edition 2023, ChenomX Inc., Edmonton, AB, Canada). Abbreviation: GABA, γ-aminobutyrate. Spectra were acquired using the WASTED-II pulse sequence [34] with irradiation at δ = 4.68 ppm (pulse power of 3.8 dB; pulse duration of 7.5 μs).

Added [Cu(II)] (µmol/L)	Resonance(s) (ppm)	Biomolecule ^1^H NMR Assignment
7.1	1.33 (*d*) and 4.13 (*q*)	Lactate-CH_3_ and -CH, respectively
	1.48 (*d*)	Alanine-CH_3_
	2.06 (*s*) and 1.82 (*dd*)	N-Acetylneuraminate-NHCOCH_3_ and -C_3_H (β), respectively
	3.25 (*t*) and 3.42 (*t*)	Taurine-CH_2_NH_3_^+^ and ^−^O_3_SCH_2_, respectively
	3.56 (*s*)	Glycine-CH_2_
	7.08 (*s*) and 7.93 (*s*)	Histidine-C4H and -C2H imidazole ring protons
	6.88 (*d*) and 7.23 (*d*)	Tyrosine-β-CH_2_ and α-CH
	7.32 (*m*), 7.37 (*m*), and 7.43 (*m*)	Phenylalanine-C_2_H/C_6_H, -C_4_H, and -C_3_H/C_5_H, respectively
	8.46 (*s*)	Formate-H
14	2.41 (*s*)	Succinate-CH_2_s
28	2.51 (*s*)	Methylamine-N(CH_3_)
	2.76 (*s*)	Dimethylamine-N(CH_3_)_2_
	2.91 (*s*)	Trimethylamine-N(CH_3_)_3_
42	1.05 (*t*) and 2.17 (*q*)	Propionate-CH_3_ and -CH_2_, respectively
	1.64 (*tq*)	*n*-Butyrate-β-CH_2_
	2.39 (s)	Pyruvate-CH_3_
	1.89 (*m*), 2.29 (*t*) and 3.00 (*t*)	GABA-β-, α- and γ-CH_2_, respectively
54	3.20 (*s*)	Choline-N(CH_3_)_3_
67	1.19 (*t*) and 3.66 (*q*)	Ethanol-CH_3_ and -CH_2_-, respectively.
0–67: No change	1.11 (*d*)	iso-Butyl alcohol-CH_3_s

Since not all Cu(II) ion-mediated changes were clearly distinguishable from direct visual examination of the ^1^H NMR profiles alone, both univariate and multivariate (MV) metabolomics analysis was implemented to elucidate further spectral modifications (as outlined in Section 2.3 below).

With regard to Figure 2, it is important to note that the 4-hydroxyphenylacetate-H2/H6 doublet resonance (δ = 7.16 ppm) overlaps significantly with that of the corresponding signal of tyrosine, so the superimposed signal appears to be a partial triplet.

**Figure 3:**
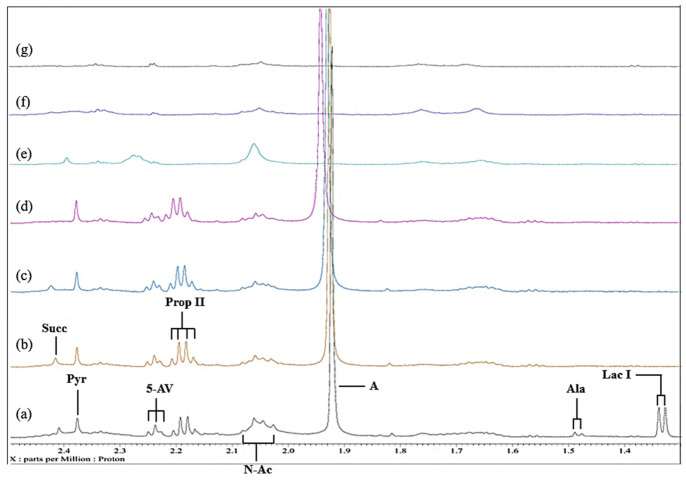
Expanded 1.30–2.50 ppm regions of the 600.17 MHz ^1^H NMR spectra of (**a**) a control human salivary supernatant specimen, and the same sample following equilibration with Cu(II) at added final concentrations of (**b**) 7.1, (**c**) 14, (**d**) 28, (**e**) 42, (**f**) 54 and (**g**) 67 µmol/L. Typical spectra are shown. Abbreviations: as in Figure 1.

Figure 3 shows the expanded 1.30–2.50 ppm region of the ^1^H NMR spectra acquired on a typical WMSS sample treated with a stepwise increase in concentration of Cu(II). Similar to those identified in Figure 1 and Figure 2, all spectral modifications in the high-field region outlined above were observable in this series of spectra too, most notably as line broadenings.

### 2.3. Univariate and MV Metabolomics Analysis of WMSS-Cu(II) ^1^H NMR Titration Datasets

#### 2.3.1. ANOVA Results

**Figure 4:**
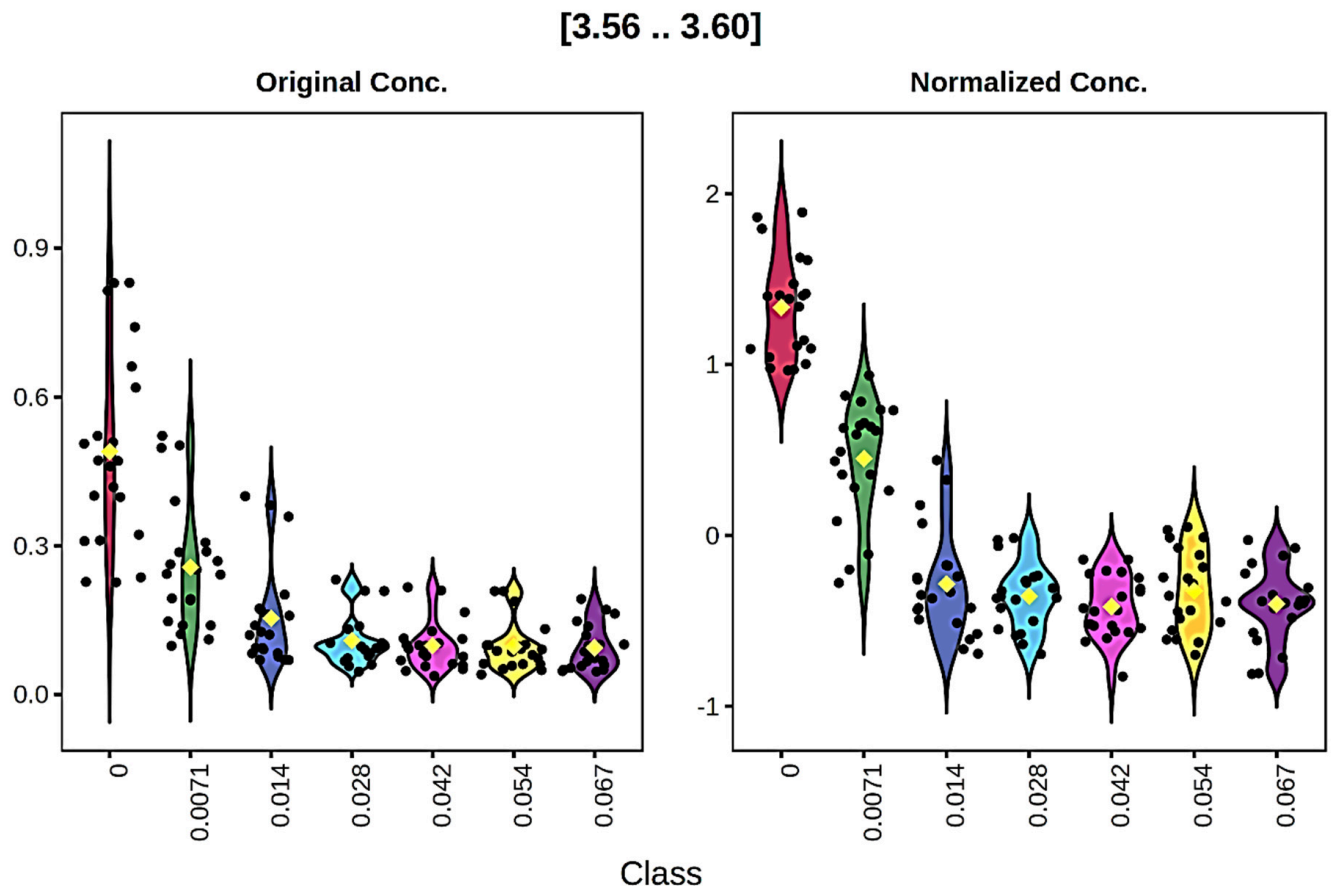

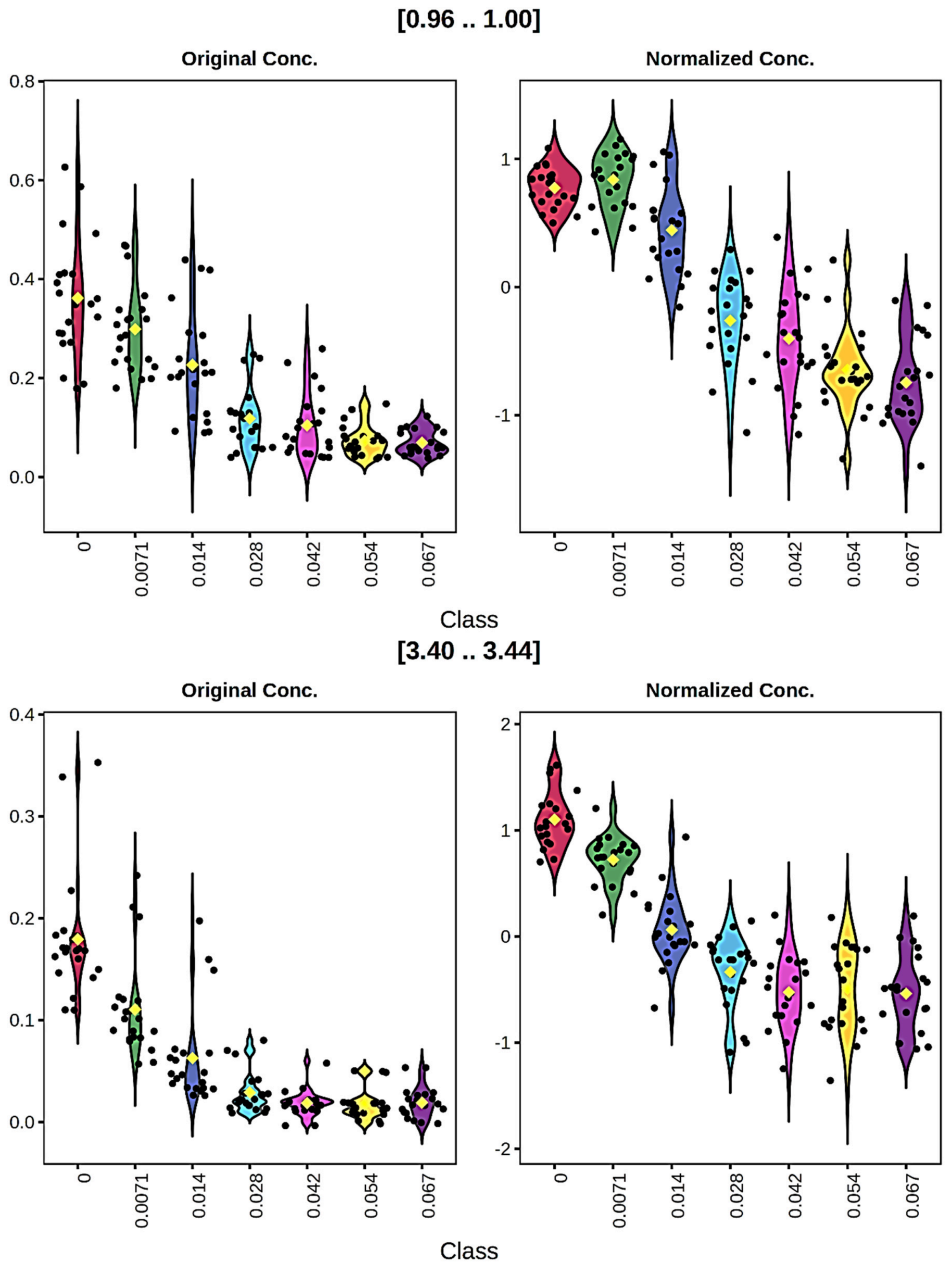

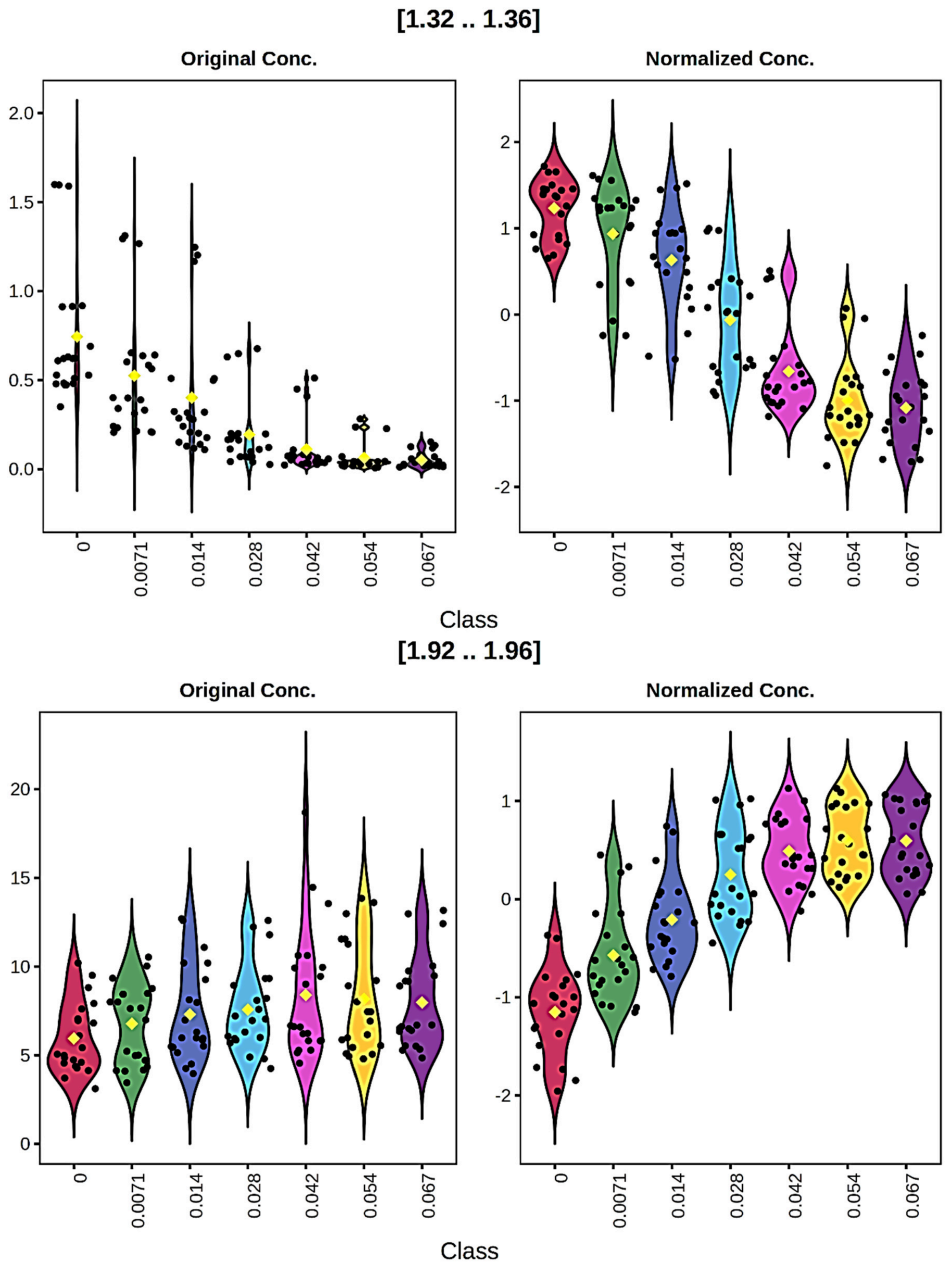

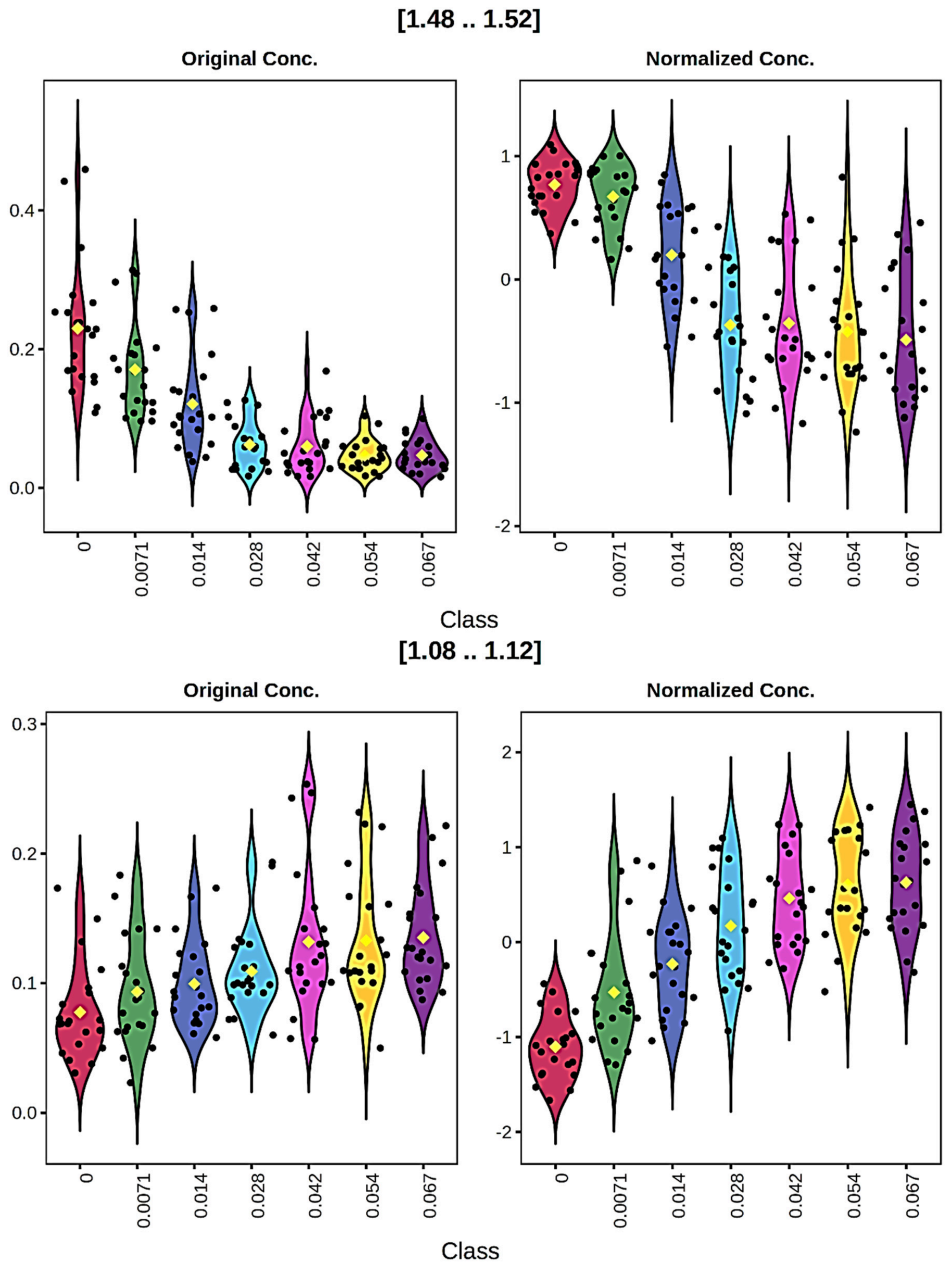

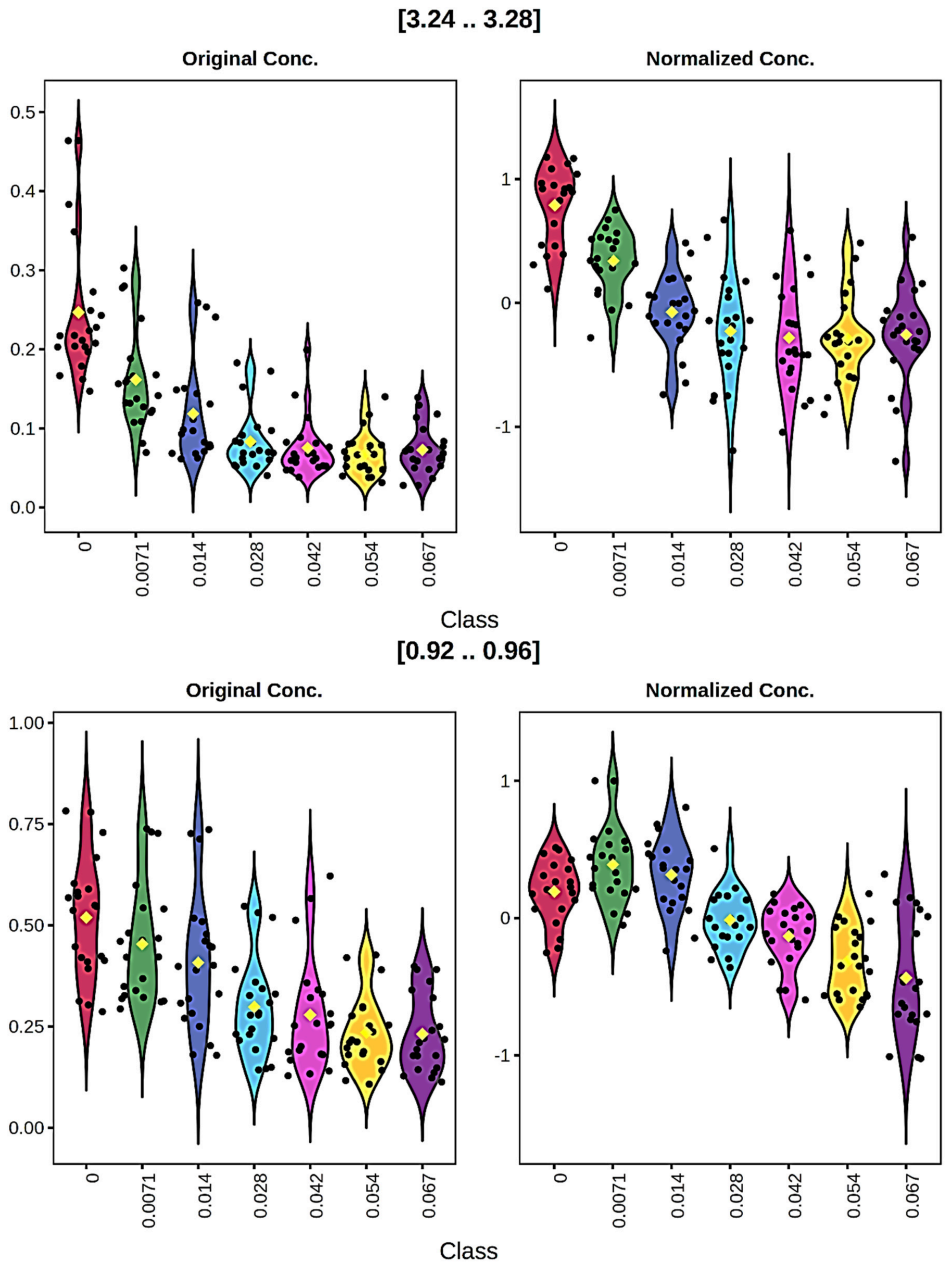
Plots of “raw” (left-hand-side) and normalised (right-hand-side) ^1^H NMR fixed-frequency width signal intensities ([2.80 … 2.84] (top) and [4.00 … 4.04] (bottom), 2.80–4.00 and 4.00–4.04 ppm buckets, respectively) for WMSS samples titrated with increasing concentrations of Cu(II). These data show examples of added “concentration lags”, which occur in the ranges of 0–0.0071 and 0–0.028 mmol/L, respectively, for these buckets. Datasets were constant sum-normalised (CSN), variance homogenisation-transformed and Pareto-scaled prior to analysis. Different colours correspond to different added Cu(II) concentrations, specifically, red, 0.00; green, 7.1; dark blue, 14.0; turquoise, 28.0; pink, 42.0; yellow, 54.0; and purple, 67.0 µmol/L (equivalent to 0.00–0.067 mmol/L, as indicated).

One-way completely randomised design ANOVA was applied to evaluate the statistical significance of the replicate titrations performed with added Cu(II) (7.1–67 µmol/L), and *p* values for the statistical significance of each ^1^H NMR bucket within the ^1^H NMR spectral profile range of 0.80–8.48 ppm in Figure 1 and Figure 2 were determined for the CSN-normalised dataset. Results acquired revealed the most important metal ion-induced modifications to WMSS ^1^H NMRs, and the orders of these and their FDR-corrected *p* values are detailed in Table 2. However, it was clear that on consideration of these modifications, the most significant decreases observed were those for glycine, leucine, taurine, lactate and alanine in that order, whereas the most significant increases in resonance intensities found were those of acetate, valine and glutamine (examples only), and corresponding violin plots for each of these univariate changes are shown in Figure 4.

#### 2.3.2. AHC-Supported Heatmap Analysis

**Figure 5:**
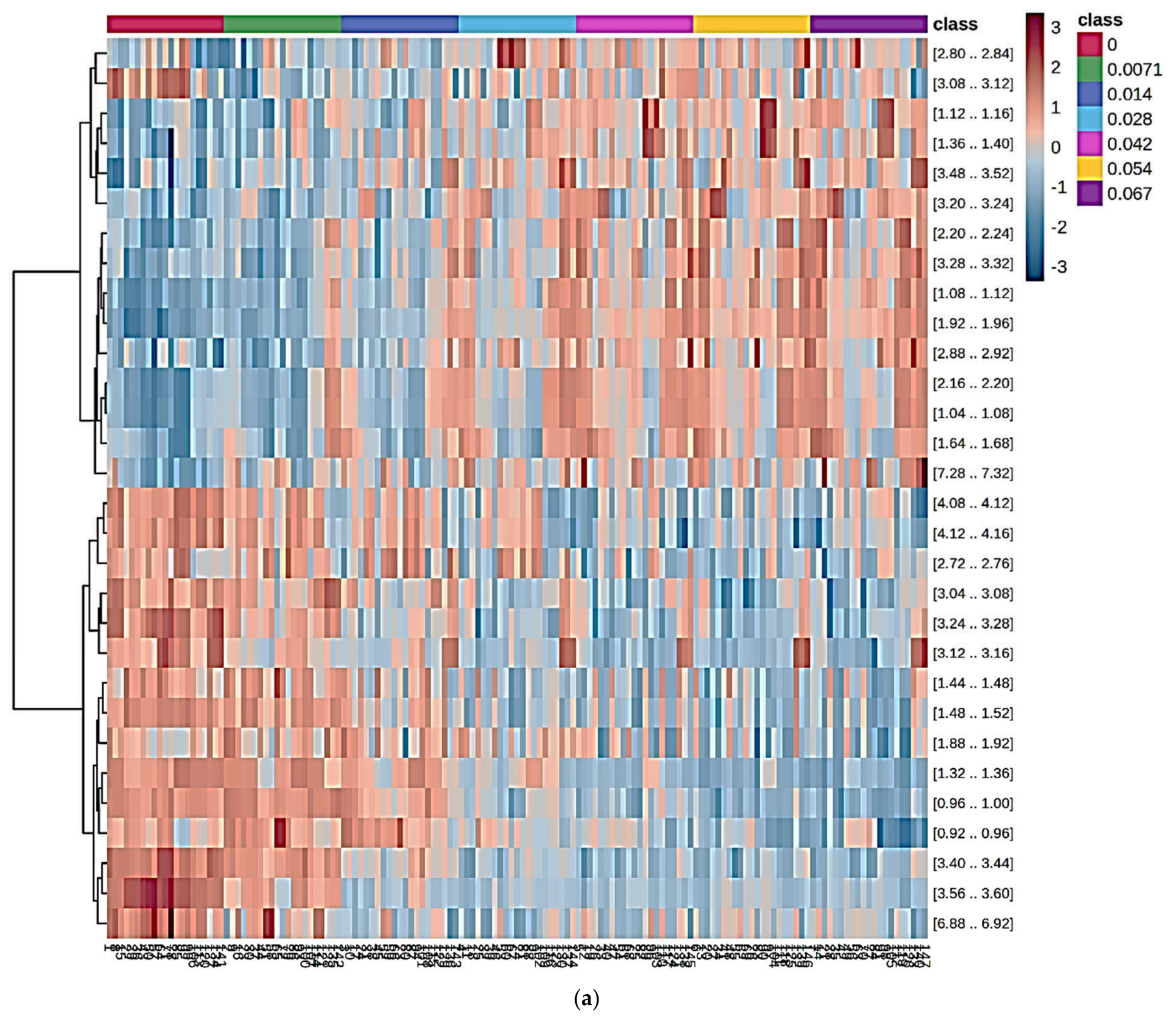

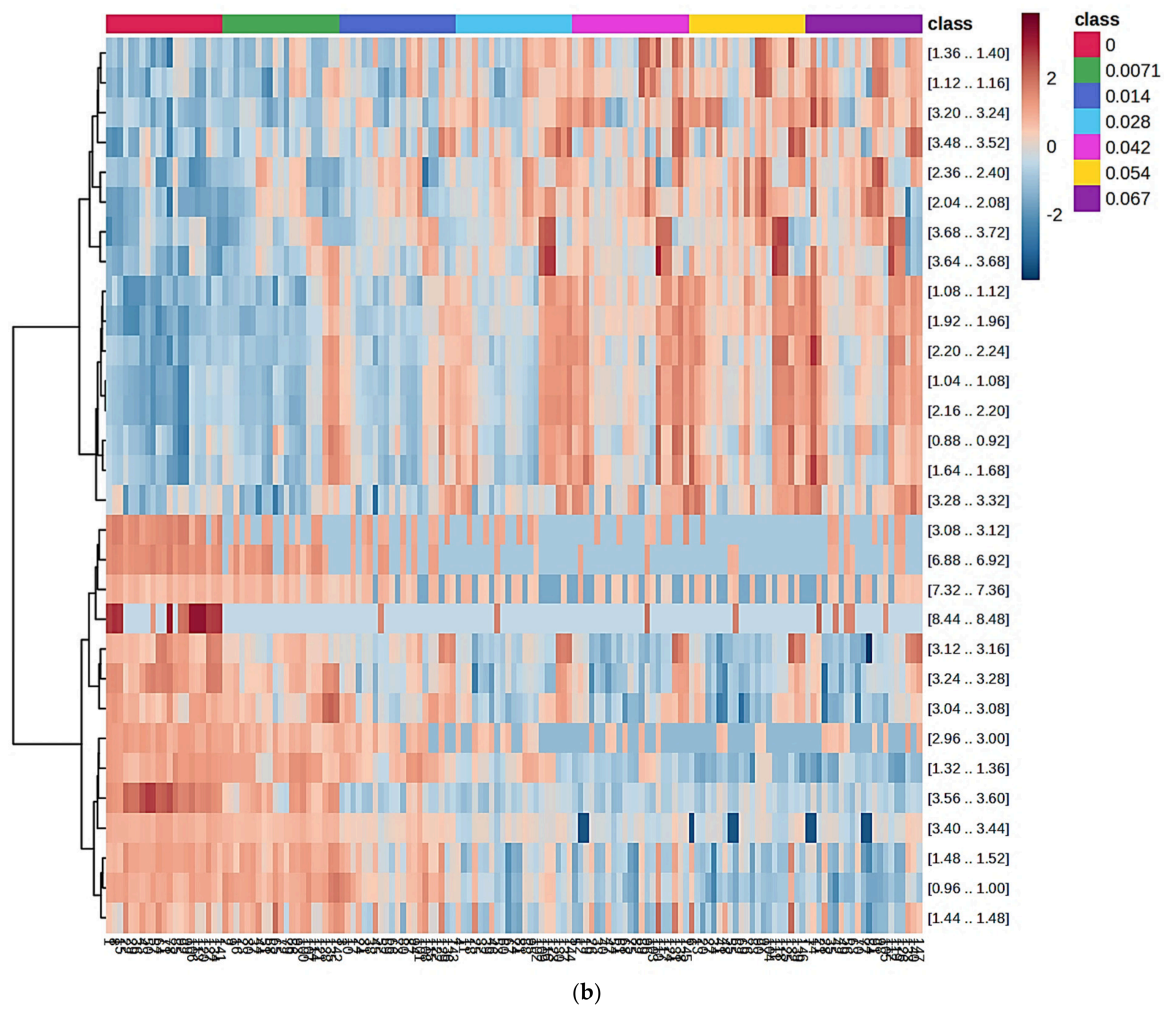
AHC-assisted heatmaps showing the top 30 WMSS sample 0.04 ppm width chemical shift buckets and their changes in intensities in response to added Cu(II) concentrations (0, 0.0071, 0.014, 0.028, 0.042, 0.054 and 0.067 mmol/L), as indicated on the far right-hand-side ordinate axis. The left-hand-side ordinate axis shows AHC of the 0.04 ppm buckets, showing clear concentration-dependent decreases in intensity for the bottom 15 variables, and corresponding concentration-dependent increases in the intensities of the top 15 buckets. Results shown are those obtained using (**a**) CSN (**top**) and (**b**) PQN (**bottom**) row-wise normalisation approaches.

An improved cross-spectral visualisation of these changes can be observed in Figure 5, which shows the heatmap diagram for added Cu(II) ion-dependent modifications to the intensities of the top 30 bucket variables. Clearly, two types of changes were observed: distinct decreases in intensity from 7.1 to 14–28 µmol/L Cu(II) (the lower 15 buckets featured), or clearly visible increases from 14, but more predominantly from 28 µmol/L Cu(II) (the higher 15 buckets). It therefore seems very likely that subsequent to the Cu(II)-mediated consumption of resonances attributable to glycine, leucine, taurine and lactate, the intensities of some largely unaffected signals increase in view of the prior disappearance or partial disappearance of a number of higher intensity WMSS resonances, e.g., those of glycine, taurine and mainly lactate. This is markedly the case in situations where the constant sum-normalisation (CSN) approach was employed. This analysis was supported by the application of AHC (left-hand-side ordinate axis), which showed that the dataset was split into two major clusters, each of which is split into two sub-clusters. Hence, the decrease and increase in intensities observed for the above two heatmap groups correspond exactly to the two major AHC clusters, lower and upper, respectively. The two sub-cluster sets ran upwards on the left-hand-side ordinate scale from [6.88 .. 6.92] to [1.44 .. 1.48], [3.12 .. 3.16] to [4.08 .. 4.12] ppm (lower major cluster), [7.28 .. 7.32] to [2.20 .. 2.24], and [3.20–3.24] to 2.80 .. 2.84] ppm (upper major cluster).

Also clear was that when the intensities of the lower left-hand-side cluster of 15 buckets had been depleted by added Cu(II), those of the upper left-hand-side cluster commenced their increase in intensity, a phenomenon again fully consistent with the above heatmap. Since they are of relatively low intensity, the aromatic signals did not feature strongly in this discriminatory dataset; however, FDR-adjusted *p* values obtained for the two histidine imidazole ring signals were found to be ≤2.0 × 10^−5^. Repeats of this clustering work using differing row-wise normalisation techniques were also performed, and interestingly, we found that use of median- or no row-wise normalisation applied at all also gave rise to this same pragmatic ‘early-decrease’ or ‘late-increase’ Cu(II) concentration-patterned distinction between these two sets of responses (Figures S1.1 and S.1.2, respectively). This AHC analysis was again repeated, but this time using the Product Quotient Normalisation (PQN) strategy, which employed the median bucket intensity values from the untreated zero-control WMSS spectral profiles, and this, as a conglomerate spectrum, served as the row-wise normalisation standard in this case. The results from this alternative analysis are now shown at the end of Section 2.3.2, and experimental details concerning this are outlined at the end of Section 4.7.2 of this paper.

When using the PQN procedure in place of the CSN row-wise normalisation, again, the complete dataset was found to split into two major AHC clusters (together with two sub-clusters for each one), the first featuring sequential decreases in intensities, the second corresponding decreases. ^1^H NMR buckets included in these two different clusters were similar to those found with CSN, although, as expected, the sequence of these differed somewhat for both cluster sets. Indeed, these were Isoleucine-γ-CH, leucine-CH_3_, alanine-CH_3_, taurine-CH_2_SO_3_^−^, tlycine-CH_2_, lactate-CH_3_, 2.99–3.00 ppm, Creatinine-N(CH_3_)/Lysine-ε-CH_2_, Taurine-CH_2_NH_3_^+^, 3.12–3.16 ppm, formate-H, phenylalanine- and tyrosine-aromatic signals, and the 3.08–3.12 ppm bucket (14 metabolites/buckets in total) for the lower intensity decrease cluster, and trimethylamine N-oxide-(CH_3_)_3_, *n*-butyrate-CH_2_, 0.88–0.92 ppm, propionate-CH_2_/glutamine-β-CH_2_, valine-CH_3_, Acetone-CH_3_, acetate-CH_3_, 1.08–1.12 ppm, 3.64–3.68 ppm, 3.68–3.72 ppm, N-acetylsugar/glycoprotein-NHCOCH_3_, pyruvate-CH_3_, 3.48–3.52 ppm, Choline-N(CH_3_), *iso*-Propanol-CH_3_, and acetoin-CH_3_s (16 metabolites in total) for the upper intensity increase one. Hence, overall, there was a similarity between the results acquired from conducting the AHC analysis using two different row-wise normalisation methods.

Interestingly, it should be noted that selected, relatively high-intensity buckets within the lower intensity decreasing bucket clusters of these AHC diagrams, for example those ascribable to the lactate-CH_3_ and Taurine-CH_2_NH_3_^+^, also had buckets located immediately downfield which were featured in the upper, intensity increasing cluster, i.e., the 1.36–1.40 and the 3.28–3.32 ppm buckets. These results are fully consistent with the participant-dependent intensity shifts to immediately downfield buckets as described below.

### 2.4. Cu(II)- and Participant-Dependent Downfield Shifts in Selected WMSS Biomolecule Resonances

A two-factor randomised blocks analysis-of-variance (ANOVA) model was employed in order to ascertain differential added Cu(II) ion-mediated responses to a series of some of the more intense WMSS resonances for which curious downfield shifts were observed for at least some of the participants’ WMSS samples evaluated. Notably, this model also included a first-order added Cu(II) ion x participant interaction effect, which was tested for its statistical significance.

In order to investigate this intriguing effect in more detail, we selected a series of six major salivary ^1^H NMR metabolite buckets (all of 0.04 ppm frequency width), along with those located immediately downfield of them. We then took the spectral profile intensities of each of these downfield buckets and divided them by those of their immediately upfield ones in each case. Further to this, the ratios of the intensities of these ‘downfield’ buckets to those of their adjacent ‘upfield’ ones were determined, and these values were then analysed using a two-way randomised blocks analysis-of-variance (ANOVA) model. In this manner, we further investigated this upfield shift effect by making covariate adjustments for determining the statistical significance of the ‘between-added Cu(II) ion concentrations’ source of variation for the above major ^1^H NMR bucket variables. This was conducted both in the presence and absence of the potentially confounding ‘between-participants’ component of variance in this model, and also with the ‘between-added Ni(II) level’ x ‘between-participants’ first-order interaction effect considered.

The relatively intense key WMSS resonances tested according to this model were the 1.04–1.08 ppm propionate-CH_3_, the 1.32–1.36 ppm lactate-CH_3_, the 1.48–1.52 alanine-CH_3_, the 1.92–1.96 acetate-CH_3_, the 2.04–2.08 N-acetylsugar-CH_3_, and the 2.40–2.44 ppm succinate-CH_2_ group signals (the pyruvate-CH_3_ 2.36–2.40 ppm bucket was excluded in view of the overlap of the immediately downfield one with that of the 2.40–2.44 ppm succinate-CH_2_ group). Corresponding immediate downfield buckets considered for each of these metabolite ISB values were 1.08–1.12, 1.36–1.40, 1.52–1.56, 1.96–2.00, 2.08–2.12 and 2.44–2.48 ppm, respectively. The results obtained are now visible in the Table below, which is now Table 3 in our revised manuscript.

These results clearly show that for these more intense WMSS resonances, the added Cu(II) ion variable influenced this effect for all signals investigated, but for three of them (i.e., those of alanine-CH_3_, lactate-CH_3_ and propionate-CH_3_), there was also very highly statistically significant ‘between-participant’ effects, and this provides evidence that this curious added Cu(II)-dependent downfield shift was also dependent on the participant WMSS profiles. Moreover, the first-order interaction was also statistically significant for the alanine-CH_3_ group, and this indicates a non-additive response of this effect for this signal.

We also computed simple Pearson’s correlations between the above ‘nearest-neighbour’ bucket ratios and added Cu(II) ion concentrations, and found that those for the -CH_3_ groups of propionate (*p* = 1.21 × 10^−7^), lactate (*p* = 4.56 × 10^−16^), and acetate (*p* = 7.38 × 10^−7^), and that of the succinate-CH_2_ group (*p* = 1.55 × 10^−3^), were all very highly positively significant, whereas that for the N-acetylsugar-CH_3_ signal was not. However, interestingly, that for the alanine-CH_3_ group was marginally negatively correlated (r = −0.353).

### 2.5. Investigation of ^1^H NMR Line-Widths at Half-Height (HHW or Δv_1/2_) in WMSS Samples

In order to investigate the influence of added Cu(II) ion on the HHW values of WMSS biomolecules, we measured the latter parameter for a number of resonances, but these were limited to those where this was possible, since many of the resonances which were clearly detectable in the ^1^H NMR profiles of control (untreated) WMSS samples broaden quite substantially early on in the spectral titration, with significant numbers of them not being clearly visible at or past these points, as shown in Figure 1 and Figure 2. For some of the more intense WMSS signals, it was, however, possible to monitor their HHW value up to an added level of 42 µmol/L, whereas, for others, such as that of the succinate-CH_2_ group singlet only, this parameter could only be monitored at added Cu(II) ion values of up to 14 µmol/L.

Two strategies were therefore selected to investigate the HHW values of WMSS resonances: Firstly, all participant sample buckets were normalised to those of their blank zero-control WMSSs.

**Figure 6:**
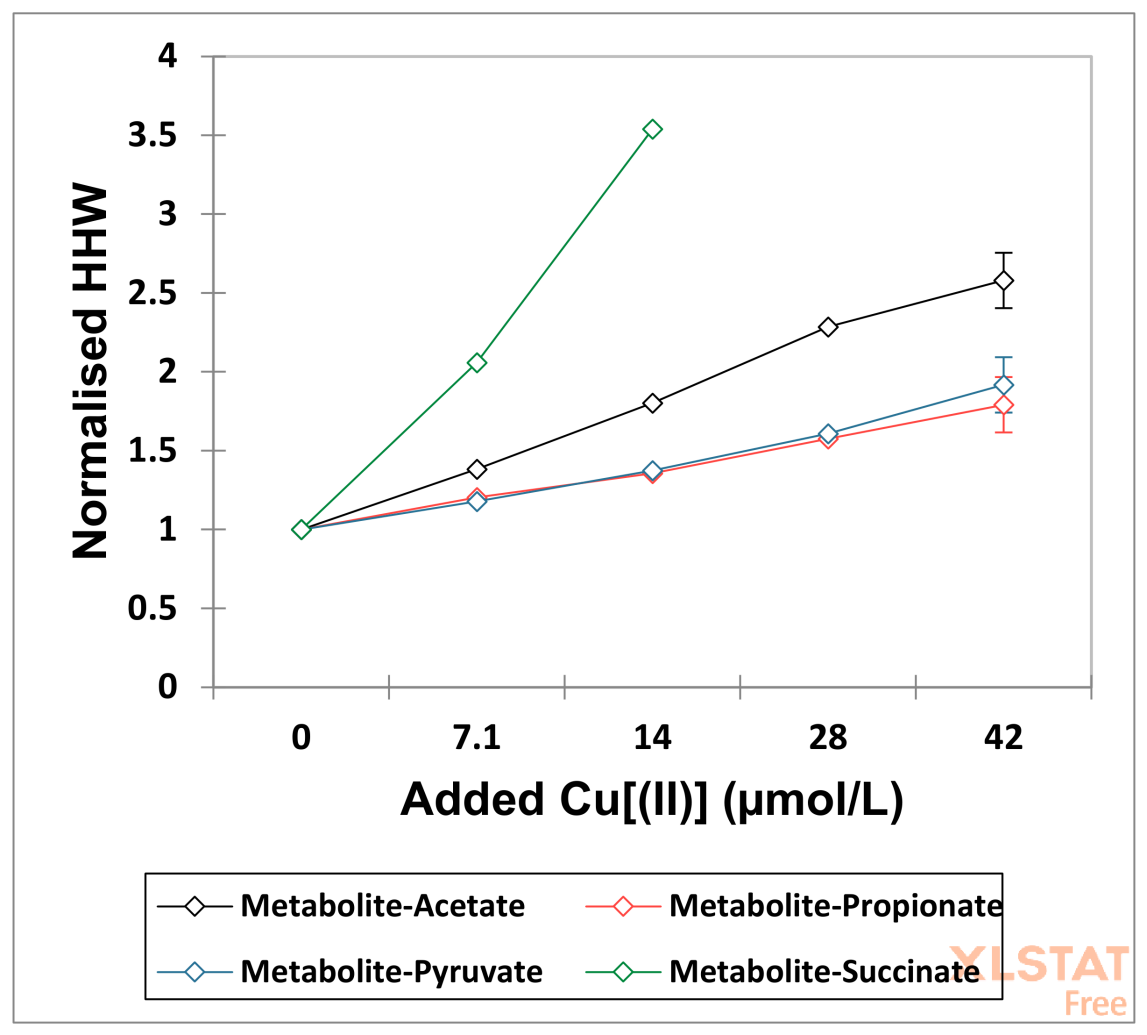
ANOVA-derived plots of normalised mean HHW ± 95% CIs for metabolite resonances versus added Cu(II) ions for spectral titrations of WMSS samples (n = 12 replicates) with Cu(II) up to concentrations of 42 µmol/L for acetate, propionate and pyruvate, and up to 14 µmol/L only for succinate (broadenings beyond these limits prevented determinations of HHW values for each resonance). All data points were normalised to the mean values of their zero-control WMSS samples, so that all values at an added Cu(II) ion level of 0.00 are represented as 1.00.

HHW values were then computed for the propionate-CH_3_, lactate-CH_3_, pyruvate-CH_3_ and succinate-CH_2_ resonances, and these values were then subjected to ANOVA analysis using a two-factor randomised blocks model, with ‘between-added Cu(II) ions’, ‘between-metabolites’ and their first-order interaction effect as major sources of variation, along with fundamental (unexplained) error. This analysis not only confirmed very highly significant differences ‘between added Cu(II) ion concentrations’ (*p* = 1.93 × 10^−19^), but also ‘between metabolites’ (*p* = 1.56 × 10^−9^). Moreover, the two-factor interaction effect was also significant (*p* = 1.82 × 10^−7^). Hence, these results clearly confirm differential responses of the HHW parameter for each metabolite tested, with acetate giving rise to a very significantly greater spectral response than those of propionate and pyruvate, and which remains substantially lower than that observed with the dicarboxylate anion succinate (Figure 6).

Secondly, we computed simple Pearson’s linear regression between the WMSS metabolite HHW values and the added Cu(II) ion concentrations, and found that although the regression coefficients of these plots were similar for both propionate and pyruvate (1.348 ± 0.064 and 1.398 ± 0.065, respectively (mean ± 95% CI)), these values for acetate and succinate were both found to be very significantly greater, i.e., 1.74 ± 0.15 and 2.013 ± 0.38, respectively. Hence, again, this provides good confirmatory evidence for the differential HHW parameter responses towards added Cu(II) for these metabolites.

From an examination of the Cu(II) ion titration spectra acquired, it is quite clear that some resonances broaden more or much more so than others (when normalised to the zero-control samples). For example, it is very clear that the pyruvate resonance at 2.388 ppm (singlet) broadens much less so than that of the succinate singlet (2.410 ppm), and this is also observable from the sequential titration data now shown in Figure 6.

Although a ‘generalised broadening’ could also be related to static field inhomogeneity, the degree of broadening was checked for many of the resonances investigated. Indeed, the wide range of quantitative data acquired supported the observation that this effect is certainly not effectively the same for all resonances. Therefore, it was possible to unambiguously ascribe it to direct and selective interactions between the ^1^H NMR-detectable biomolecules and added Cu(II) ions.

In our recent paper focused on salivary Ni(II) ion speciation [28], we were able to compare ^1^H NMR intensities which were normalised to that of TSP, but this was only possible for direct comparisons of the zero-control WMSS samples with those from the group containing only the lowest concentration of added Ni(II) (71 µmol/L), since this low Ni(II) level group was the only one which did not give rise to any statistically significant TSP resonance broadening effects.

However, in this case, with added Cu(II) in place of Ni(II) ions, albeit at 10-fold lower added concentrations than those featured in the Ni(II) study, unfortunately, significant increases in line-broadening at half-height (HHW) were detected even at the lowest added Cu(II) level (only 7.1 µmol/L). Indeed, results from a two-way randomised blocks ANOVA model demonstrated that both the ‘between-added Cu(II) levels’ and ‘between-participants’ effects were substantially significant (*p* = 9.63 × 10^−17^ and 5.44 × 10^−8^, respectively). Moreover, post hoc analysis using the Bonferroni approach showed that there were highly significant differences between the zero control and all other added Cu(II) ion concentration groups, with levels ranging from 7.1 to 67 µmol/L. A bar diagram plot of mean HHW value versus added Cu(II) level ± 95% CI is shown in Figure S2.1.

### 2.6. SEM Analysis of Cu(II)-WMSS Deposits

#### 2.6.1. Particulate Morphology

**Figure 7:**
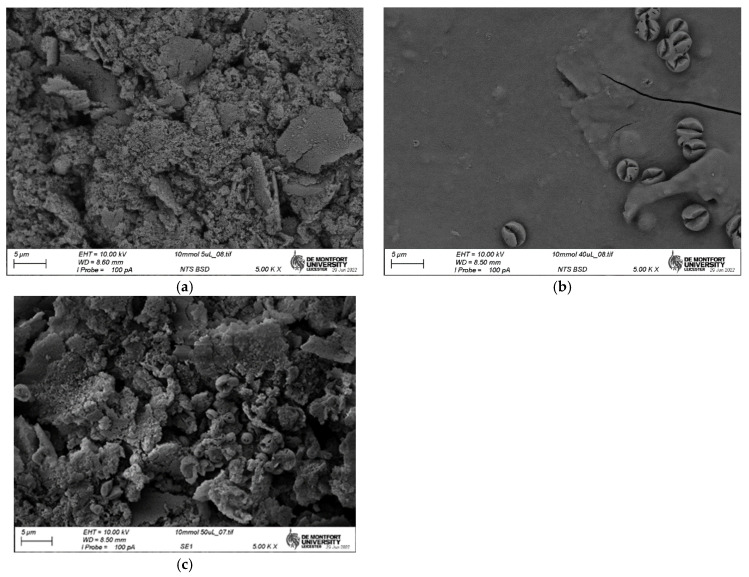
Morphology of Cu(II)-WMSS deposit samples: (**a**) circular Cu grains arising from the preliminary addition of 5 µL of a 10.00 mmol/L solution of Cu(II)_(aq.)_. (**b**,**c**) Agglomerated Cu grains derived from the addition of 40 and 50 µL, respectively, of a 10.00 mmol/L solution of Cu(II)_(aq.)_.

Cu(II)-WMSS precipitate samples were analysed by SEM/EDS, and, primarily, the morphology of the samples was observed. All samples were crystalline in appearance, but with some notable differences; the images for the sample primarily treated with 5 µL of a 10.00 mmol/L Cu(II)_(aq.)_ solution (final added concentration 71 µmol/L) showed dense crystalline material at low magnification, although at higher magnification it was observed that crystalline material was an agglomeration of copper grains of varying sizes. Samples pre-treated with 10, 20, 30 and 40 µL of a 10.00 mmol/L solution showed smooth circular particles of approximately 1 µM in size. Figure 7a,b show the type of morphology and the agglomerated particles observed for two classes of these samples, respectively.

At the higher added volume of 50 µL of the 10.00 mmol/L Cu(II)_(aq.)_ stock solution, agglomerated crystalline material was observed, with some circular nanoparticles visible amongst the flakes (Figure 7). However, precipitate samples generated from only a 1.00 mmol/L stock concentration of Cu(II)_(aq.)_ were found to consist of dispersed small circular particles, again with the higher volume of 50 µL added from this solution showing a much denser concentration of particles.

#### 2.6.2. Elemental Composition of the Cu(II) Deposits

Determination of the elemental compositions of the added Cu(II)-induced salivary deposits was subsequently performed by SEM/EDS analysis. These results (Table 4) confirmed the successful formation of Cu(II)-biomolecule and/or inorganic anion complexes or chelates with predominant elements of copper (18.09 ± 5.77 (mean ± SD), range 10.03–32.36%); carbon (34.09 ± 9.69%, range 11.10–45.93%); oxygen (29.23 ± 4.55%, range 22.80–36.13%); nitrogen (8.35 ± 2.41%, range 5.26–12.86%); phosphorous (3.01 ± 2.10%, range 1.30–9.46%); and sulphur (2.57 ± 2.01%, range 1.00–8.13%) by weight. The intense Cu and C peaks yield a [C]:[Cu] elemental content ratio of approximately 10:1, and this indicates that the deposit may comprise a copper(II)-carbonate nanoparticulate species, since the atomic percentage of copper is often found to be relatively low to that of carbon in such species. Moreover, in studies of CuO/Cu_2_O or carbonate-based nanostructures, copper(II)-carbonate–hydroxide serves as a common intermediate [35,36].

**Figure 8:**
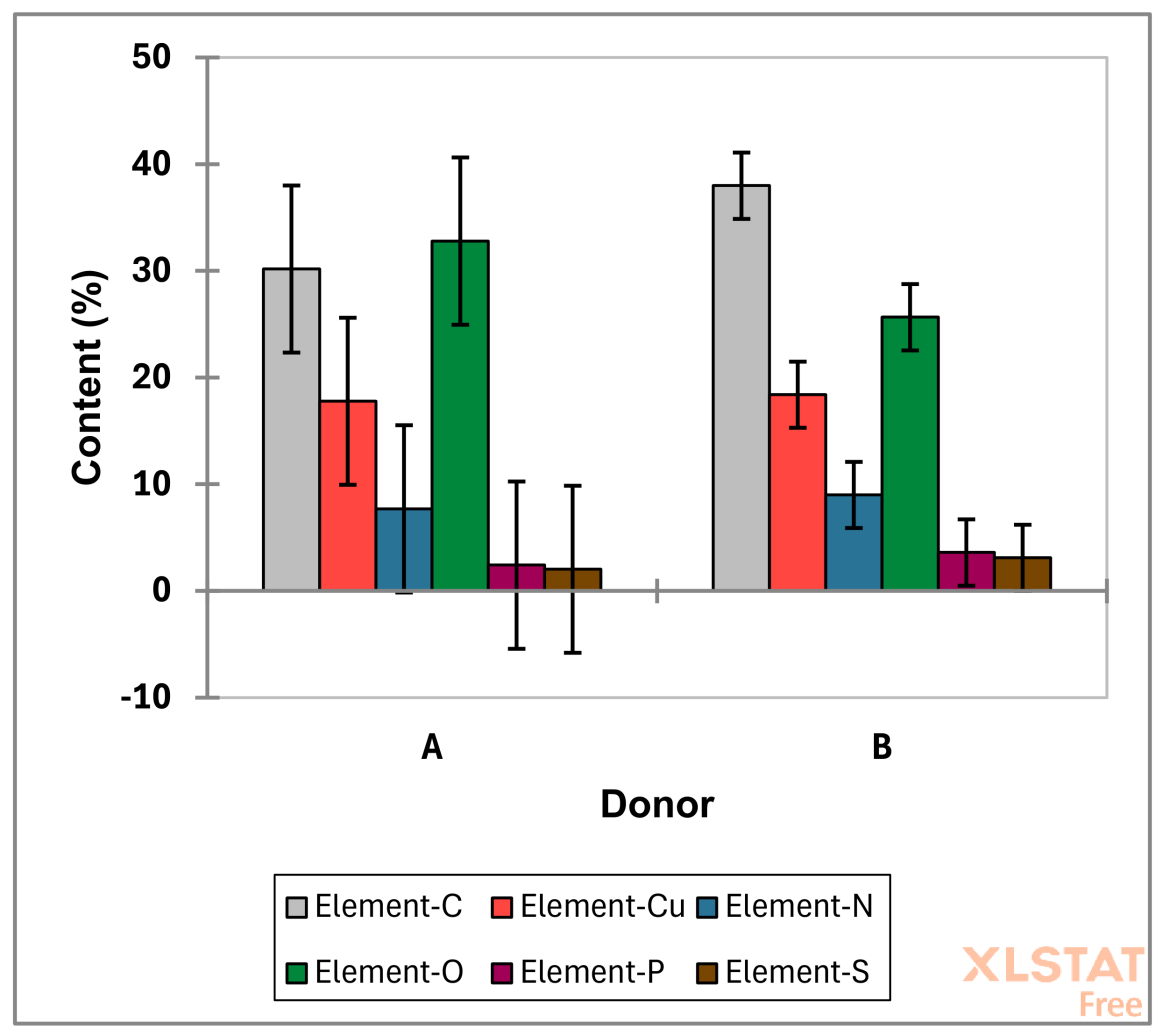
Mean ± 95% CI percentage elemental compositions of Cu, C, N, O, P and S for n = 5 Cu(II) ion-induced deposit samples obtained from a series of WMSS samples donated by two individual human participants (A and B). Elements are colour-coded as shown.

Figure 8 shows the mean % elemental composition of Cu, C, O, N, P and S for a total of n = 6 separate deposits/precipitates arising from the separate addition of Cu(II) ions to WMSS samples collected from two different human participants. Although there was no significant difference found between the two participants for all elements tested using the constant sum-row normalisation method, this diagram clearly reveals that the % order of elemental composition of these deposits was the same for both donors, with its carbon content being greater than that of Cu(II).

### 2.7. Correlations Between the Elemental Compositions of Cu(II)-Induced WMSS Deposits with and Without Consideration of the Added Cu(II) Level Variable

For the Cu(II)-induced particulate solid mass dataset alone, we found statistically significant negative correlations between its carbon and nitrogen contents, and that of Cu (r = −0.6038 (*p* = 0.038) and −0.5771 (*p* = 0.041), respectively). Moreover, we also found a highly significant positive correlation between the N and C contents of the precipitate (r = 0.836, *p* = 0.00069). These data indicate that the deposit formed contains amino acids, peptides and/or proteins, and/or alternative biomolecules with related C and N contents such as purines and pyrimidines. However, it appears that these contents are not positively connected to those of added Cu, so, possibly, this metal ion is independently precipitated as inorganic species, perhaps as oxides, hydroxides and/or carbonates. The reduction in Cu(II) to Cu(I) to form brown-coloured Cu_2_O species, perhaps as nanoparticulates, however, cannot be ruled out, in view of the presence of low levels of electron-donating thiols, e.g., cysteine, and also phenolics such as the amino acid tyrosine. However, in culture medium [29], this reaction system is largely mediated by high concentrations of the reducing sugar glucose, which is present at approximately 10 mmol/L in this matrix, but certainly not so in saliva.

Nevertheless, we also monitored correlations between the added Cu(II) level (total) and that of the deposit’s elemental constitution. From these investigations, we found a strong positive correlation between the deposit’s % Cu content and its added Cu level (r = 0.783, *p* = 0.0026). Moreover, as observed for correlations between elemental compositions in the isolated precipitate alone, there were quite strong negative r values between the added Cu(II) concentration and the deposit percentages of C (r = −0.758, *p* = 0.0043) and N (r = −0.709, *p* = 0.0098), and this again verifies the singularity (independence) of its Cu content from that of potential salivary metabolites such as amino acids and proteins as complexant species therein. Further molecular identification studies of the deposit formed were undertaken by FTIR-ATR spectroscopy, and these are outlined below.

### 2.8. FTIR-ATR Analysis of WMSS and Insoluble Cu(II)-Induced Deposits or Aggregates Derived Therefrom

**Figure 9:**
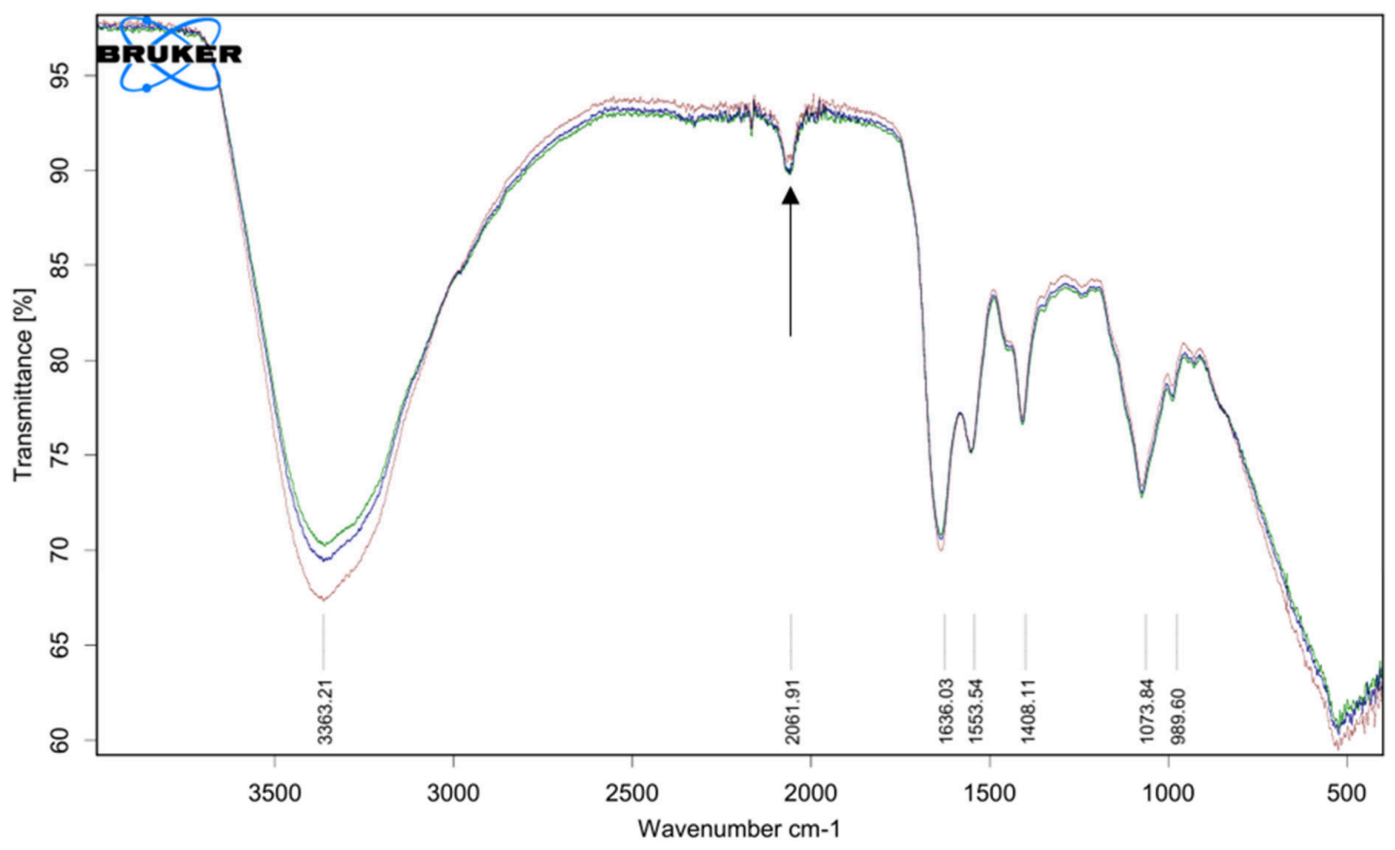
FTIR-ATR spectral profiles acquired on dried thin films of an untreated, control WMSS sample (n = 3 replicate samples collected from the same participant, coloured mauve, blue and green). The arrow indicates the thiocyanate anion -C≡N stretching band. Full assignments for this spectrum are provided in the legend to Figure 10 below.

Figure 9 displays typical FTIR-ATR spectra derived from n = 3 replicates of an untreated WMSS specimen collected from a healthy participant. This spectrum arises from a range of different biomolecules and ions therein, and, hence, a variety of diverse absorption bands are observable; these are assignable to proteins, lipids, nucleic acids and thiocyanate anion. Indeed, it displays previously reported characteristic absorption bands visible for nucleic acids (1100–850 cm^−1^), in which the P=O asymmetrical and symmetrical stretching vibrations from the -PO_2_ phosphodiester groups of phosphorylated biomolecules were notable (1073 cm^−1^) [37], although it is expected that the nucleic acid bands would predominantly arise from the release of DNA and RNA species from lysed cells. Notably, the removal of microbes, cells and debris from samples prior to WMSS preparation via the centrifugation step involved may indeed ensure removal of much of the DNA present in original samples. Moreover, fasted saliva specimens contain N-acetylated glycoproteins, including the molecularly mobile carbohydrate side-chains of these species and general glycosylated proteins, such as α-amylase, and these may be detectable after the >8 h abstention period required prior to collection of the 0.00 h time-point sample. Since they have an absorption maxima within the 1080–950 cm^−1^ range, this enzyme may also be partially responsible for the absorption band centred at 990 cm^−1^. A quite sharp band centred at 2061 cm^−1^ is also notable, and this is ascribable to the -C≡N stretch of thiocyanate anion (SCN^−^).

**Figure 10:**
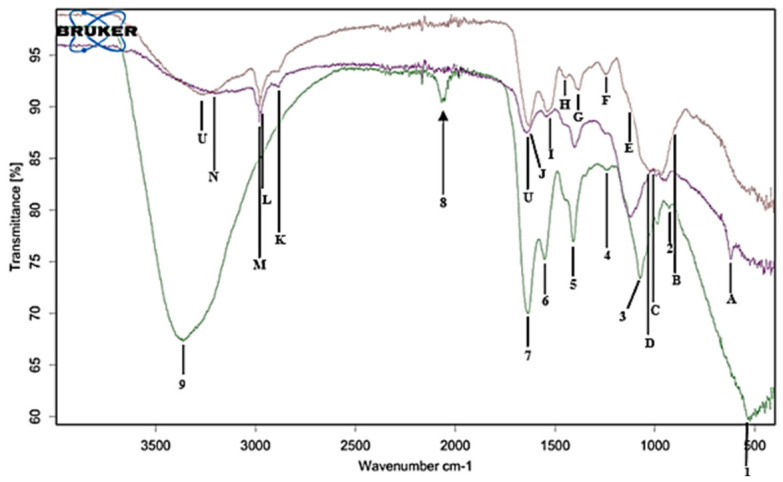
FTIR-ATR spectra of thin films of typical untreated WMSS specimen (green spectrum), the precipitate formed from the reaction of added Cu(II) ion to it followed by washing with ice-cold water (brown spectrum), and that of the residual WMSS specimen following removal of the added Cu(II) ion-induced precipitate (mauve spectrum). Three replicate samples were collected from the same participant. The arrow indicates the thiocyanate anion -C≡N stretching band. Letter and numerical assignments correspond to those available in Table 5.

In Figure 10, the mauve-coloured profile corresponds to the thin film of a residual WMSS specimen obtained following added Cu(II)-induced precipitation, and then removal of this deposit via centrifugation; analysis of this resulting precipitate is depicted as the brown spectrum. This was derived from the same intact control WMSS sample giving rise to the spectra depicted in Figure 9, which is displayed here as a green profile.

Upon comparison of the WMSS specimens before and after Cu(II) addition, a marked depletion of FTIR-ATR absorption bands is clear in the purple spectrum. Indeed, this was a reproducible observation for all WMSS samples subjected to this bioanalytical protocol. Following Cu(II) deposit generation, the WMSS bands located at 989, 1073, 1048, 1553, 1636 and, most distinctively, 3363 cm^−1^, were virtually all removed. Furthermore, the loss of the -SCN^−^ band (centred at 2061 cm^−1^) is also of much interest. As expected, absorption bands were markedly weaker in the FTIR-ATR spectra acquired on those of the biomolecularly depleted samples derived from Cu(II)-treated WMSS samples than those in their corresponding untreated controls. Indeed, these observations are consistent with quite a substantial uptake of a wide range of WMSS biomolecules by solid Cu(II) deposits/aggregates.

FTIR-ATR analysis of the deposits formed from treating replicate WMSS samples with Cu(II) (Figure 9 and Figure 10) revealed notable similarities and differences between these samples and their respective untreated control samples, and results acquired are tabulated in Table 5, with differences in absorbance and displacements in functional group band wavenumber enabling the two types of profiles to be readily distinguishable. This Table provides a full range of biomolecular assignments that are likely, or at least possible, although in view of the many possible contributions towards each frequency band, an extensive level of superimposition is expected. Indeed, these differences inferred the occurrence of major structural modifications arising from Cu(II) addition to WMSS.

When analysed as a dry thin film, an absorption band located at 3363 cm^−1^ (predominately the Amide A *v*(N-H) assignment) was shifted to 3263 cm^−1^ (bands 9 and N, respectively) in the deposited solid matrix. Examples of other displacements that can be observed include that of the protein *vs*(-CH_3_) band at 1408 cm^−1^ (band 5), the Amide I band located at 1636 cm^−1^ (band 7), and the Amide II band at 1553 cm^−1^ (band 6), which were displaced to 1381 cm^−1^ (band G), 1631 cm^−1^ (band J), and 1542 cm^−1^ (band I), respectively. In overview, the absorption bands acquired from FTIR-ATR analysis of the Cu(II) precipitate are largely derived from protein, amino acid amide (C=O, C-N, C-H, C-C, O-H, N-) and phosphate species, in addition to fatty acids and lipids, the presence of which may stem from the surface of any aggregates or nanoparticulates formed. Intriguingly, following the removal of the Cu(II)-induced precipitate, only very limited quantities of SCN^−^ anion remained in the resulting depleted WMSS matrix.

As expected, all spectra acquired contained water vibrational bands located at ca. 3290 cm^−1^ (O-H stretching, predominantly in the untreated WMSS profile) and 1634 cm^−1^ (H-O-H bending) [37].

### 2.9. Reversal of Cu(II)-Induced ^1^H NMR Spectroscopic Modifications with Added EDTA

**Figure 11:**
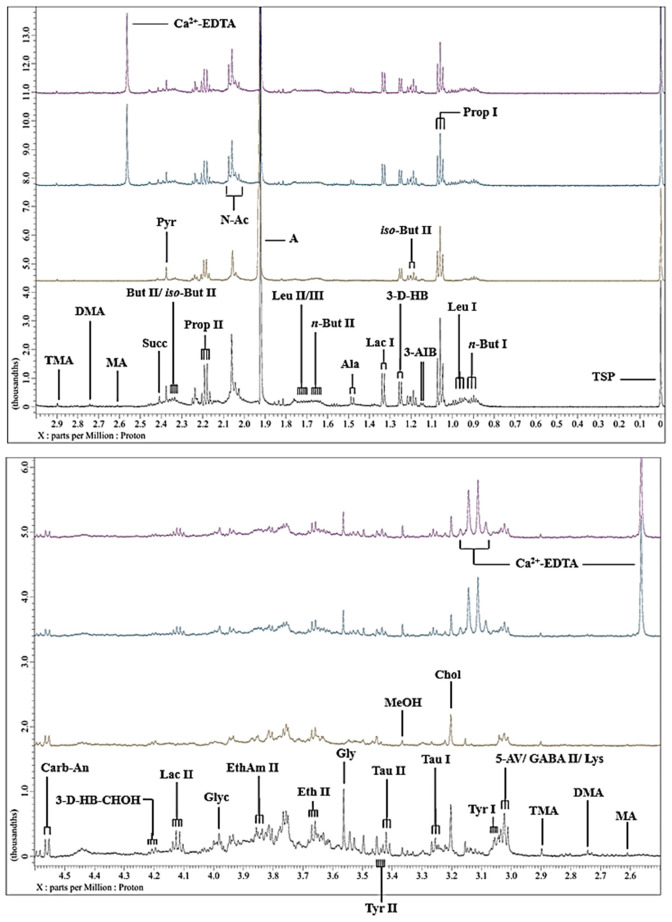

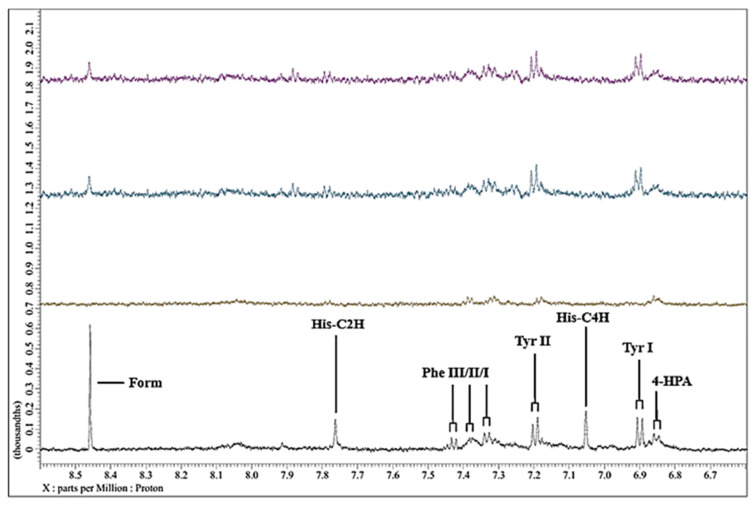
Influence of added EDTA (770 µmol/L) on the ^1^H NMR spectra of WMSS samples following treatment with a final Cu(II) concentration of only 670 nmol/L. (**Top**–**bottom**) Expanded 0.00–3.00, 2.50–4.60 and 6.60–8.60 ppm regions of 600 MHz WASTED ^1^H NMR profiles, respectively. Ordered bottom-to-top spectral profiles in each diagram comprise those of untreated culture medium; as the bottom spectrum, but after spiking the sample with 670 nmol/L Cu(II)_(aq.)_; second from top, as the previous (lower) spectrum, but following the addition of 770 µmol/L EDTA (spectrum obtained 30 min. following addition); and top, as the previous spectrum, but after acquiring it 24 h post-EDTA treatment. Typical spectra are shown; different spectral profiles have different colours. Abbreviations: as in Figure 1, Figure 2 and Figure 3, with EDTA, Cu^2+^-EDTA and Ca^2+^-EDTA representing 1H NMR resonanes of EDTA, and its 1:1 copper and calcium ion complexes.

The Cu(II)-mediated resonance broadenings noted above should, in principle, be reversible, assuming that sufficient amounts of this metal ion remain in the aqueous phase of WMSS samples. We therefore added the powerful hexadentate chelator EDTA to Cu(II)-treated WMSSs subsequent to the removal of any deposit generated immediately after addition of Cu(II). The investigators therefore added a final concentration of only 670 nmol/L Cu(II) to WMSS samples and, even at this low added level, observed substantial broadenings of a range of resonances, both aliphatic (lactate, succinate, alanine, methylamines, etc.; see Figure 11 top and middle), and aromatic (notably the two imidazole ring protons of L-histidine, together with tyrosine’s two doublets; see Figure 11 bottom). Nevertheless, following the addition of an excess of EDTA (770 µmol/L), all of the above ^1^H NMR signals reappeared in the spectral profiles. These spectra were acquired at time-points of 0.5 and 24.0 h post-EDTA addition, although no significant time-dependence of the intensities of these signals was notable. However, at least some of these regenerated resonances appeared to be broader than those of the untreated (control) WMSS samples, an observation suggesting that at least some Cu(II) remains coordinated to these biomolecules following EDTA addition. Furthermore, the ^1^H NMR signals of tyrosine and phenylalanine, which were primarily broadened by added Cu(II), reappeared in spectra obtained at both these time-points. The aliphatic biomolecule resonances (for example, those of lactate and alanine, etc.) were also found to return to the profiles acquired prior to Cu(II) ion addition. These exchange processes are very thermodynamically favourable, with a high stoichiometric, thermodynamic equilibrium constant (Keq), and, therefore, this experiment confirms EDTA’s powerful Cu(II) ion-chelating ability.

Following the addition of excess EDTA, a signal attributable to its 1:1 Ca^2+^ ion complex (ethylenic proton singlet at δ = 2.57, and -NCH_2_CO_2_^−^ proton AB coupling pattern resonance at δ = 3.06–3.17 ppm) became visible in spectra, together with those of the uncomplexed EDTA chelator at ca. 3.2 and 3.6 ppm, respectively.

## 3. Discussion

This study represents the first comprehensive ^1^H NMR-based investigation of Cu(II) ion speciation in human WMSS samples, providing molecular-level insights into the interactions between Cu(II) and salivary biomolecules. Indeed, our findings demonstrate that Cu(II) ions engage in diverse and concentration-dependent binding interactions with multiple salivary constituents, resulting in characteristic spectroscopic ‘signatures’ that reflect the complex coordination chemistry occurring in the oral environment. These results have important implications for understanding the bioavailability, sensory perception, and toxicological properties of copper(II) ions encountered through dietary sources and drinking water.

### 3.1. Cu(II) Ion-Induced Modifications to Salivary ^1^H NMR Spectra

The addition of Cu(II) ions to WMSSs induced pronounced alterations in their ^1^H NMR spectroscopic profiles, which were largely influenced by the Cu(II) ion level added and consistent with the formation of paramagnetic Cu(II)-biomolecule complexes. This paramagnetic metal ion possesses a single unpaired electron that generates a local magnetic field, leading to paramagnetic relaxation enhancement (PRE) of adjacent nuclear spins. This phenomenon manifests as selective line broadening and, in extreme cases, complete signal attenuation for protons, especially those in close proximity to the paramagnetic centre(s).

Observations of broadened and diminished resonances from specific amino acids, peptides, organic acid anions and other small molecules indicate direct coordination of these species to Cu(II). The concentration-dependent nature of these effects suggests the presence of binding sites with varying affinities for this metal ion. Indeed, at lower Cu(II) concentrations, selective broadening of signals from high-affinity donor ligands (such as histidine and glutamine, along with peptides and proteins containing these residues) would be expected, while at higher concentrations, additional binding to lower-affinity sites on other biomolecules, such as those with carboxylate anion O-donors and different proteins, becomes apparent. Indeed, MV hierarchical clustering analysis of these data revealed that there were two major classes of added Cu(II)-mediated changes observed (Figure 5), the first specifically involving marked intensity reductions from added levels of 7.1 to 14–28 µmol/L Cu(II), with the second featuring clearly visible intensity rises from 14, but more predominantly from 28 µmol/L Cu(II); these two classes reflected the lower and higher 15 metabolite modifications shown in Figure 5. It therefore seems very likely that subsequent to the Cu(II)-mediated paramagnetic ion-mediated consumption of signals ascribable to relatively high-intensity WMSS biomolecules of high affinity for added Cu(II), e.g., those of glycine, leucine, taurine and mainly lactate, the intensities of others, with lower Cu(II) affinity, increase from the CSN approach employed in this analysis. Hence, the second class of resonances showing Cu(II) ion-dependent intensity increases in Figure 5 (top 15 bucket resonances on the left-hand side of Figure 5) is likely to arise from the prior full or partial disappearance of some of the above higher intensity WMSS resonances in the first class of complexants described.

The identity of the affected signals provides insight into the specific complexation partners of Cu(II) in this biofluid. Amino acids with side-chains capable of metal ion coordination, particularly those containing nitrogen (histidine, lysine and arginine, etc.), and oxygen (aspartate, glutamate, serine and threonine, etc.) donor atoms, represent prime candidates for primary, non-protein Cu(II) complexation [18].

Notably, Cu(II) ions can form salivary complexes with free histidine, which is a well-established chelator for this metal ion, forming stable *bis*– and *tris*–histidine complexes at neutral pH values through coordination involving the imidazole nitrogen and, depending on pH, the free amino and carboxylate groups [38]. Cysteine and methionine can also coordinate to Cu ions through their sulphur donor atoms, although cysteine’s thiol group has a much higher affinity for soft metal ions in their reduced state (Cu(I)) compared to their harder (Cu(II)) ions) [39]. Nonetheless, at elevated copper ion concentrations or under partially reducing conditions in saliva, cysteine–copper(I) ion interactions could, at least in principle, also contribute towards any ^1^H NMR changes observed.

Thermodynamic redox potentials for copper(II)/(I)–amino acid complexes (e.g., those of glycine and alanine) typically range from ca. 0.0 to +0.3 V vs. NHE (normal hydrogen electrode), with these parameters being highly dependent on pH, concentration, and overall ligand/chelator structure. Frequently, such complexes demonstrate a quasi-reversible single-electron reduction from Cu(II) to Cu(I) in aqueous solution [40,41,42,43]. Some key facets of the redox behaviour of Cu(II)/Cu(I) complexes of amino acids and further biomolecules are that (1) usually, 1:1 metal ion–amino acid ligand complexes have a higher level of reactivity than the more stable 1:2 complexes; and (2) as expected, these redox potentials are critically dependent on pH–Cu(II)-binding level increases with increasing pH, a phenomenon rendering electron transfer more difficult.

These redox potentials typically range from −1.26 to +0.50 V, and also depend on complex geometry, donor atoms and overall ligand structure; indeed, square planar or elongated octahedral geometries stabilise Cu(II), whereas tetrahedral geometries stabilise Cu(I) [44]. Ligands with N- and S-donor atoms significantly affect the redox potential, with, as we might expect, Cu(I) favouring sulphur and Cu(II) having a preference for N. Moreover, with regard to geometrical effects, tetrahedral or flattened tetrahedral complexes often have higher positive redox potentials (with a more oxidising Cu(II) ion as the metal ion centre), and octahedral or tetragonally elongated structures mainly show negative, lower redox potentials, with more “reducing” Cu(II) ions [41].

Typical example redox potentials are +0.153 V for Cu(II)_(aq.)_/Cu(I)_(aq.)_, and −0.21 for the α-synuclein-Cu(II)/Cu(I) system. Notably, the anodic dissolution of Cu(II) complexes of glycine, valine and alanine shows redox potentials ranging from −0.20 to +0.15 V (vs. Ag/AgCl) [44], and, therefore, in general, the complexation of added Cu(II) ions by WMSS biomolecules such as amino acids (and proteins) lowers the redox potential of the Cu(II)/Cu(I) couple, a process which facilitates its participation in Fenton or Fenton-like reactions in vivo (Equation (1)).

Cu(I) + H_2_O_2_ → Cu(II) + ^●^OH + OH^−^ (1)

The reversibility of Cu(II)-induced spectroscopic modifications upon addition of EDTA provides strong evidence that the observed ^1^H NMR effects arise from specific Cu(II)-biomolecule coordination rather than non-specific aggregation or precipitation. EDTA effectively and strongly chelates Cu(II) ions, stripping them from lower affinity biomolecular binding sites and at least partially restoring the native ^1^H NMR spectral profile of salivary biomolecules. This reversibility confirms the dynamic nature of Cu(II) ion binding in WMSSs, and suggests that its speciation is governed by thermodynamic equilibria amongst multiple competing ligands.

### 3.2. FTIR-ATR Spectroscopic Evidence for Cu(II)-Biomolecule Interactions and the Cu(II)-Mediated Deposition of Precipitates Formed in WMSSs

Complementary FTIR-ATR analysis revealed the detection of amide I and amide II bands of salivary proteins present in the precipitate arising following Cu(II) addition, along with some significant alterations to their spectra. These changes may be partially consistent with metal ion-induced perturbations of protein secondary structure and/or their direct coordination to amide groups. The amide I band (primarily C=O stretching vibrations of the peptide backbone, ~1630–1680 cm^−1^) and amide II band (N-H bending coupled with C-N stretching, ~1520–1560 cm^−1^) are highly sensitive to protein conformation. Shifts in the position, intensity, or breadth of these bands can indicate changes in α-helix, β-sheet, or random coil content, or, alternatively, reflect alterations in the hydrogen bonding environment of the amide groups.

The observed FTIR-ATR changes suggest that the binding of added Cu(II) induces conformational rearrangements in salivary proteins, potentially exposing or sequestering hydrophobic regions and altering protein–protein or protein–ligand interactions. Additionally, direct coordination of Cu(II) to carboxylate anion oxygen atoms or amide nitrogen atoms would perturb the electron density distribution around these groups, leading to shifts in the corresponding vibrational frequencies.

The formation of insoluble copper(II)–protein complexes at higher added concentrations, as evidenced by increased turbidity and precipitate formation, is consistent with previous reports identifying specific salivary proteins (of molecular masses ~29 kDa and ~33 kDa) that form insoluble aggregates with added Cu(II) [24]. These proteins may undergo cross-linking or polymerisation reactions mediated by Cu(II) ions acting as bridging ligands between multiple protein molecules. Such aggregation could contribute to the astringent mouthfeel associated with elevated copper levels in drinking water, since protein precipitation reduces the lubricating capacity of saliva and may interact with oral epithelial surfaces.

However, in view of the very low concentration of proteins in human saliva in general, it certainly appears that the precipitate formed following addition of Cu(II) ion to WMSS samples contains selective amino acids, and/or inorganic species such as Cu(II)-oxides, -hydroxides, carbonates and phosphates, etc., in addition to low protein contents, as indicated herein.

Interestingly, in 2002, Marsia et al. [45] performed FTIR analysis of prion protein repeats of marsupial source. In addition to major modifications to the secondary structure and an enhancement of H-bonding revealed by a red shift of >7 cm^−1^, changes to its tertiary structure on copper ion-binding were found via the development of a new absorption band at 1564 cm^−1^, and this arose from a histidine ring vibration. This copper ion-mediated conformational modification occurred at pH > 7.0 and was pH-dependent. Moreover, a ca. 5-fold decrease in amide function hydrogen–deuterium exchange was noted for the peptide reacting with added Cu(II) ions.

### 3.3. Implications for Copper Ion Bioavailability and Toxicity

The speciation of Cu(II) ions in human saliva has direct implications for its bioavailability and potential toxicity. Free, hydrated Cu(II) ions, along with a wide range of small soluble copper complexes, are generally considered to be more bioavailable for absorption across mucosal membranes than larger protein-bound or precipitated forms. Indeed, the findings obtained in the current paper indicate that at physiological salivary pH and at copper concentrations relevant to dietary intake (typically <2 mg/L in drinking water) [9], a substantial fraction of it becomes linked through binding to low-molecular-mass salivary biomolecules. Notably, these complexation processes may serve protective functions, which could mitigate potential cytotoxic effects [46]. Moreover, recently, the redox activity of copper ions has been suggested to act as a major adverse factor regarding the evolution of neurodegenerative diseases, e.g., Parkinson’s, Alzheimer’s, and amyotrophic lateral sclerosis via the anomalous liberation of ‘catalytic’ copper ions into the intracellular space [47].

However, it appears to the authors of the current paper that there remains a lot of confusion regarding the true chemical nature of the species which are responsible for catalysing or promoting the adverse generation of reactive oxygen species (ROS), such as superoxide anion (O2^●−^), hydrogen peroxide (H_2_O_2_) or hydroxyl (^●^OH) radical in vivo, and many researchers and clinicians seem to attribute this function to the so-called **“free”** Cu(II)aq./Cu(I)aq., species, and **not** to any other form of it which counts or is described as **“bound”** copper ions. Unfortunately, this fails to make sense, since with the exception of certain complexes which may have their redox activities limited by unfavourable redox potentials or their aqueous solubilities, all have the ability to exert significant, often positive effects towards the production of ROS. Indeed, amino acid ligands such as L-histidine and L-glycine significantly reduce the redox potentials of the Cu(II)/Cu(I) couples from those of the Cu(II)aq./Cu(I)aq. couples [43,44], and, therefore, when the metal ion centre is in the reduced form, participation in the Fenton reaction involving oxidation of Cu(I) to Cu(II) (Equation (1)) will occur under physiological or ambient laboratory conditions, with this effect being approximately inversely proportional to the decrease in redox potential. The same is true for protein-bound Cu(I) or Cu(II) species, although in these cases any escaping ^●^OH radical is likely to be scavenged by localised polypeptide residues.

Nevertheless, it is also of much importance to note that although selected biological ligands can diminish the above redox potential, they also have the capacity to “site-specifically” scavenge any ^●^OH radical generated from the Cu(I) centre [48], and, therefore, they may indeed serve to protect physiological environments against ROS-mediated oxidative damage, most notably those in the oral location.

However, it has been speculated that at higher Cu concentrations, such as those that might result from the consumption of contaminated drinking water, the leaching of copper plumbing in newly constructed buildings, and/or exposure to copper-containing dental materials, the capacity of salivary binding sites for this metal ion may become further saturated. Many of these low-molecular-mass Cu complexes are capable of initiating or propagating oxidative stress. Indeed, as noted above, their participation in Fenton or Fenton-like reactions can generate aggressively reactive ^●^OH radicals that may directly damage an array of biomolecules, including lipids, proteins, and nucleic acids [49] (Equation (1)). Cu(I) ions can arise from dietary Cu(II) through the actions of endogenous or dietary reductants such as ascorbate and thiols, etc. Intriguingly, in the current study, we found that the addition of only 7.1 µmol/L, or even sub-micromolar levels of Cu(II), gave rise to the complete removal of the histidine imidazole ring signals, and this result is largely consistent with those of Hong et al. [18], who found that at added concentrations of ≤10 mg/L (equivalent to 157 µmol/L), 50–70% remained liquid-soluble and in the solution phase. However, in contrast, we also found that there was still a significant level of precipitate formation accompanying the addition of levels of Cu(II) ≥ 7.1 µmol/L.

Such Cu(II) complexes formed can trigger oxidative stress, alter enzyme functionality, and restrict nutrient availability. Indeed, when complexed by physiological ligands, copper ions can disrupt normal cellular processes, which can adversely lead to the formation of ROS, most notably the ^●^OH radical, through Fenton reactions. This phenomenon adversely affects cell viability and function by inducing oxidative damage, ultimately leading to DNA impairment and disruptions in its repair mechanisms upon interaction with histones, which are crucial for maintaining DNA integrity.

In principle, Cu(II) can oxidise electron-donating biomolecules such as L-cysteine, resulting in a disulphide bond formation while releasing Cu(I) ions. When these reduced ions come into contact with H_2_O_2_, they can produce an ^●^OH radical (Equation (1)), reverting back to their oxidised form (Cu(II) ion within the Cu(II)/Cu(I) redox couple). This allows them to re-engage in Fenton and related reactions [50]. Numerous studies have indicated that mixtures of Cu(I) and Cu(II) with H_2_O_2_ can induce DNA damage through ROS generation, and this supports the hypothesis concerning the critical role of ROS in carcinogenic processes associated with metal particulates and metal ions [51].

Chronic exposure to excessive quantities of copper ions through drinking water has been implicated in cases of childhood liver cirrhosis, including Indian childhood cirrhosis (ICC) and Tyrolean infantile cirrhosis (TIC), both of which are associated with high copper content in water or milk prepared with contaminated water [52]. Although genetic susceptibility factors are also likely to contribute towards these conditions, the role of oral copper exposure and potential pre-systemic interactions in human saliva warrants consideration. Interestingly, understanding the speciation of salivary copper and its complexation by selected biomolecules could inform risk assessments for populations exposed to elevated copper levels.

### 3.4. Copper Speciation and Metallic Taste Perception

The perception of an adverse metallic taste from copper-containing water and foods is a well-documented phenomenon, with soluble copper species being the primary contributors to this sensory attribute [53]. Our findings that Cu(II) forms extensive complexes with salivary biomolecules may serve to provide a molecular basis for understanding the relationship between copper speciation and taste perception. Indeed, soluble, low-molecular-mass Cu(II) and/or Cu(I) complexes, such as those formed with amino acids, small peptides, or organic acid anions, are likely to be more accessible to taste receptors than large protein-bound forms or insoluble precipitates [18,19,53].

The pH-dependence of copper solubility and speciation plays a critical role in taste perception. At lower pH values (e.g., pH 5.5), copper solubility is enhanced, leading to higher concentrations of taste-active species and more intense metallic flavours [18,19]. Conversely, at near-neutral salivary pH values (6.8–7.2), copper solubility decreases, and the extent of protein binding increases, a phenomenon potentially attenuating metallic taste intensity. This pH effect may explain individual differences in copper taste sensitivity related to variations in salivary pH, or, alternatively, fluctuations in oral pH following consumption of acidic foods or beverages.

Previous studies [17,18] have suggested that different copper ion species formed in saliva have different reactivities in schemata that may be associated with the perception of metallic sensation occurring in the oral cavity. Indeed, these studies were performed to understand molecular mass-dependent copper ion speciation in human saliva, on the premise that it would help researchers to understand the perception and mechanism of metallic taste. Saliva samples were treated with Cu(II) at levels of 0, 2.5, 10, 20, or 40 mg/L as Cu in vitro (0–0.63 mmol/L). Moreover, saliva was collected before and after drinking 20 mL of 0, 2.5, and 5 mg/L Cu(II) solution in water (in vivo treatment). Copper “speciation” was operationally determined based on apparent molecular size using ultrafiltration coupled with inductively coupled plasma–mass spectrometry (ICP-MS). For in vitro copper treatment, 50–70% of copper ions were found to be soluble at Cu ≤ 10 mg/L, whereas 60–70% of it was in complex or insoluble form at added levels > 20 mg/L. For in vivo copper treatment, 90–95% of this metal ion was soluble in saliva. These results suggested that Cu(II) ions are in the soluble unbound form in saliva at low concentrations, much of this being present as complexes with low-molecular-mass metabolite ligands such as L-histidine and glutamine, for example. At higher concentrations, added Cu(II) either became insoluble or, alternatively, complexed to alternative salivary components.

### 3.5. Methodological Considerations and Future Directions

The use of ^1^H NMR spectroscopy for investigating copper ion speciation status in human WMSS samples offers several advantages over traditional analytical methods. ^1^H NMR analysis has the capacity to reveal the molecular environment and binding status of ligands for Cu(II) or Cu(I). The non-destructive nature of ^1^H NMR analysis preserves the native equilibrium of metal ion–ligand complexes, avoiding artefacts introduced by sample preparation such as filtration, dialysis, or digestion. Furthermore, this technique can easily distinguish between multiple co-existing species in solution, providing a more complete “picture” of copper ion speciation than that offered by bulk analytical techniques.

However, high-resolution ^1^H NMR analysis also has its limitations. The technique is relatively insensitive compared to mass spectrometry-based methods, requiring micromolar concentrations for the detection of minor species, although it should be noted that sub-micromolar levels are now detectable when the technique is featured in “state-of-the-art” metabolomics experiments [19]. Additionally, as expected from investigations exploring the speciation status of paramagnetic metal ions, the broadening effects induced by such added metal ions can obscure fine structural details of metal ion-bound ligands, rendering it challenging to determine precise coordination geometries or binding stoichiometries; however, such limitations may be at least partially overcome via the careful selection of added concentration ranges, which was indeed the case for the experiments featured in the current study. Complementary techniques such as electron paramagnetic resonance (EPR) spectroscopy, which directly detects the unpaired electron in Cu(II) complexes, or X-ray absorption spectroscopy (XAS), which provides information on metal ion oxidation state and coordination environment, could augment NMR findings, ^1^H-based or otherwise, in future studies.

Another important consideration is the variability of human WMSS composition amongst individuals and over time. Salivary flow rate, pH, protein concentration, and metabolite profiles can vary with circadian rhythms, hydration status, dietary intake, and oral health conditions [12,19,21]. The copper-binding capacity and speciation patterns observed in this study reflect the characteristics of unstimulated WMSS collected after an overnight fast, but this may indeed differ under other physiological conditions. Future investigations examining stimulated saliva, parotid or submandibular gland secretions separately, or those collected at different times of day, could provide a more comprehensive understanding of copper ion speciation dynamics, however.

Finally, extending these studies to in vivo settings, for example, by analysing saliva samples from individuals consuming controlled amounts of copper ions, or those fitted with copper-containing dental appliances, would allow us to investigate correlations between copper speciation patterns and physiological outcomes such as taste perception, oral health status, or systemic copper absorption. Such translational research could inform the development of strategies to mitigate ingested copper ion-related sensory and toxicological issues, including the potential formulation of copper-binding dietary supplements, or the design of safer copper alloys for dental and oral health applications.

### 3.6. Broader Implications for Trace Metal Ion Research

This study contributes to a growing body of research applying NMR-based metabolomics and metallomics approaches to investigate the speciation of trace metals in biological fluids. Previous work from this group and others has demonstrated the utility of ^1^H NMR for characterising metal–ligand interactions in blood plasma, cell culture media, and other biofluids [12,19,21,29,54]. The principles and methodologies developed for Cu(II) speciation in human saliva can be extended to other physiologically and toxicologically relevant metal ions, including zinc(II), iron(II)/(III), manganese(II), and chromium(III). Each metal ion exhibits distinct and often characteristic coordination preferences and redox chemistries, unique properties leading to characteristic speciation patterns, and biological effects.

Understanding metal ion speciation is also crucial for interpreting epidemiological and clinical data on metal/metal ion exposure and health outcomes [55]. Total metal or metal ion concentrations measured by traditional analytical methods do not necessarily reflect bioavailable or toxicologically active fractions, which critically depend on chemical speciation. By identifying the specific molecular forms of metal ions present in biological systems, researchers can develop more accurate biomarkers of exposure and effect, refine risk assessment models, and design targeted interventions to modulate their bioavailability and toxicity.

## 4. Materials and Methods

### 4.1. Sample Collection and Preparation

For this study, unstimulated human saliva specimens were collected from a total of n = 14 control adult volunteers (9 females and 5 males, mean ± SD age 25 ± 2.3 years) after an >8 h abstention period from all oral activities, including eating, drinking, smoking, and tooth-brushing to minimise the risk of “contamination” from the introduction of exogenous agents into the oral environment. Another important factor considered was the presence of any existing Cu- or other metal ion-containing dental implants that may be present, including amalgams and removable partial dentures, and participants were questioned on this prior to being invited to participate in the study. Participants were instructed to collect all “whole” saliva (ca. 4–8 mL) from the mouth into a sterile plastic universal tube once the fasting period was complete. After collection, samples were placed on ice and immediately transported to the laboratory where they were anonymised using an alphanumeric code prior to preparation. It transpired that none of the potential study participants had any of the above dental implants, and so all were recruitable to the investigation on this basis.

Whole saliva samples were centrifuged (10,000× *g* for 10 min at 4 °C) to remove cellular debris and generate whole mouth salivary supernatant (WMSS) samples. Samples not analysed the same day of collection were stored at −70 °C for a maximum duration of 30 h. An Accumet AET 15 pH metre (Fisher Scientific Ltd., Loughborough, UK) was used to determine the pH values of WMSS samples (mean ± SD pH value of 6.98 ± 0.19).

Since this study involved the use of human WMSS samples, it should be noted that all research was approved by the Research Ethics Committee of the Faculty of Health and Life Sciences, De Montfort University (DMU), Leicester (approval code no. 457249), and conducted in accordance with the guidelines outlined in the Declaration of Helsinki.

### 4.2. Treatment of WMSS Samples with Cu(II) for ^1^H NMR, FTIR-ATR and SEM Analyses

WMSS samples (0.7 mL) were spiked with Cu(II) using a 1.00 mmol/L CuSO_4_ solution prepared by dilution of a 10.00 mmol/L stock in HPLC-grade water, yielding added Cu(II) concentrations of 7.1, 14, 28, 41, 54 and 67 µmol/L. Samples were rotamixed to ensure homogenisation and centrifuged (10 min, 3000 RCF at 4 °C) to remove precipitates. Aliquots (0.54 mL) of the resulting supernatants were combined with 60 μL D_2_O containing sodium 3-trimethylsilyl [2,2,3,3-^2^H_4_] propionate (TSP; 2.90 mmol/L; Fisher Scientific Ltd., Loughborough, UK). Control samples were prepared by combining 0.54 mL of untreated WMSS with 60 μL D_2_O/TSP, which served as the chemical shift reference (δ = 0.00 ppm) and quantitative internal standard. Cu(II) addition induced the formation of a precipitate, which was isolated by centrifugation (described above), washed three times with ice-cold HPLC-grade water, and air-dried in the dark at ambient temperature (>16 h) prior to SEM and FTIR-ATR analysis.

### 4.3. ^1^H NMR Measurements

Proton (^1^H) NMR spectra were acquired employing a JEOL JNM-ECZR 600 MHz spectrometer (600.17 MHz) (JEOL UK, Welwyn Garden City, Hetrfordshire, UK) at 298 K. Spectra were collected using 256 scans (4 dummy scans) and 16,384 data points, with an acquisition time of 1.82 s, a relaxation delay (RD) of 1.0 s, and a sweep-width of 9.00 kHz. Residual H_2_O/HOD resonance suppression was achieved using the WASTED-II pulse sequence with irradiation at δ = 4.68 ppm (pulse power of 3.8 dB; pulse duration of 7.5 μs) [34].

In the NMR-based metabolomics aspects of this study, relaxation delay (RD) represents the interval between the end of an NMR acquisition (free induction decay, FID) and the beginning of the next scan. This time is critical because it enables the nuclear spins to return to their resting state aligned with the longitudinal magnetic field, a process referred to as *T*_1_, or longitudinal relaxation.

Generally, an RD value of 5 times the *T*_1_ value of slowly relaxing nuclei is considered appropriate for quantitative ^1^H NMR analysis. However, since this duration is usually more than 15–25 s, it is not optimal, nor practical, for high-throughput metabolomics experiments. Therefore, a practical compromise involves the consideration of a series of RD values between 1 and 5 s to maintain the high-throughput ratio well balanced, with a tolerable S/N ratio. Although shortening of the RD enhances efficiency, it may hinder full relaxation of nuclear spins, which results in some differences in the peak integrals due to the saturation effect. However, it remains very feasible to compare the same biomolecules between a sample set of metabolites. One improved optimisation method is to use an RD of approximately 0.8 s with an increased number of scans. This approach may improve the S/N ratio and sensitivity, especially for low-abundance metabolites. A further point which is often overlooked is that the RD value employed should always be added to the spectral acquisition time, which in our experiments was 1.82 s, in order to obtain the correct RD value. Hence, in our case, the adjusted RD value is 1.82 + 1.00 s = 2.82 s.

### 4.4. Preprocessing of Salivary ^1^H NMR Data

Baseline correction was performed using a polynomial function, Fourier-transformed, phase, zero-filling, and apodisation were applied, and chemical shifts were referenced to TSP (δ = 0.00 ppm). Data were subsequently automatically binned into 0.04 ppm-bucketing regions using ACD/Labs *Spectrus Processor 2023* (ACD/Labs, Toronto, ON, Canada) with distortions manually corrected. The residual H_2_O/HOD signal (δ = 4.65–5.06 ppm) was removed from all spectral profiles. Resonance assignments were achieved through the use of supporting databases, such as the *Human Metabolome Database* (HMDB) [34], as well as those available from the published literature [12]. For confirmation of these, *ChenomX* software (ChenomX Inc., Edmonton, AB, Canada) was employed.

### 4.5. Scanning Electron Microscopic (SEM) Analysis of Possible Cu(II)-Salivary Biomolecule Aggregates and Nanoparticulates

A Carl Zeiss (Jena, Germany) EVO LS 15 Scanning Electron Microscope equipped with an Oxford Instruments Xmax 80 mm^2^ EDS detector (Oxford Instruments, Abingdon, Oxfordshire, UK) was used to conduct EDS and SEM analysis of the Cu(II)-induced precipitate deposit. Images were taken using secondary electron (SE) and back-scattered electron (BSD) detectors, and samples were coated with 5 nm of gold, using a Quorum Q150RS Sputter Coater (Quorum, Laughton, East Sussex, UK) with an accelerating voltage of 5 kV. For energy dispersive X-ray spectroscopic (EDS) maps, samples were uncoated, and an accelerating voltage of 10 kV was used and contrasted over 20 frames.

### 4.6. FTIR-ATR Analysis of Cu(II)-Salivary Precipitate Samples

FTIR-ATR spectra were acquired using an ALPHA II-Platinum FT-IR spectrometer (Bruker UK, Coventry, UK) equipped with a platinum diamond ATR QuickSnap module. The ATR crystal was cleaned with alcohol and a background spectrum recorded prior to each measurement. Cu(II)-precipitate material formed following Cu(II) addition to WMSS samples was analysed directly on the ATR crystal. Spectra were collected over 400–4000 cm^−1^ at 2 cm^−1^ resolution with a 20 s acquisition time and atmospheric compensation; each sample was analysed in triplicate.

WMSS samples obtained after centrifugation, along with untreated controls, were also analysed by FTIR-ATR. For liquid analysis, 0.70 µL aliquots were deposited onto the ATR crystal and air-dried for ≥20 min to form a thin film prior to acquisition.

### 4.7. Statistical Analysis of Experimental Data

For both univariate and MV statistical analysis strategies applied, *MetaboAnalyst 6.0* software options were utilised.

#### 4.7.1. Univariate ANOVA Analysis of ^1^H NMR-Based Metabolomics Cu(II) Ion Titration Data

Univariate statistical analysis techniques were conducted using *MetaboAnalyst 6.0* software options (University of Alberta and National Research Council, National Institute for Nanotechnology (NINT), Edmonton, AB, Canada). For this (statistical analysis [one- factor] module), a one-way (completely randomised design) analysis-of-variance (ANOVA) model was used to determine the false discovery rate (FDR)-adjusted significance of differences between the mean ^1^H NMR bucket intensity values observed following the removal of precipitates subsequent to the addition of increasing concentrations of added Cu(II) ions (7.1–67 µmol/L added to 0.70 mL of WMSS samples as described above). The FDR-adjusted *p* values were determined, and then violin plots were produced for all those which achieved an FDR-corrected *p* value of <0.05.

#### 4.7.2. AHC Analysis of Cu(II) Ion Titration Data

Also, an agglomerative hierarchical clustering (AHC)-supported heatmap showing colour-coded individual fixed 0.04 ppm ^1^H NMR bucket responses to increasing added Cu(II) concentrations was conducted on a CSN-normalised dataset. Indeed, this approach was utilised to facilitate studies of added Cu(II) level-dependent modifications to the spectral profiles of WMSS samples, and also whether any ^1^H NMR bucket clusters found were distinctive from each other. These experiments were repeated using either no row-based normalisation, or with a median normalisation strategy. Response classes arising therefrom (classes 1 or 2) were easily decipherable.

This analysis was then repeated using an alternative row-wise normalisation strategy, namely the product quotient normalisation (PQN) approach, which employed median bucket intensities from the zero-control (no added Cu(II) ion) dataset as the normalisation standard. From these results, it was found that this PQN approach also gave rise to the generation of two major clusters with added copper(II) ion-dependent bucket intensity increases and decreases, both of which consist of two sub-clusters.

From the results acquired with this additional work, we concluded that, overall, there was a similarity between the results acquired from conducting the AHC analysis using the PQN row-wise normalisation method, and that of the CSN strategy originally applied, although, as expected, the order and extent of biomolecular contributions towards these discriminatory techniques differed somewhat.

## 5. Conclusions

This study provides the first detailed ^1^H NMR spectroscopic characterisation of Cu(II) ion speciation in human whole mouth salivary supernatants. The results acquired revealed complex and concentration-dependent interactions between this metal ion and one or more ordered series of salivary biomolecules. Findings demonstrate that Cu(II) ions complex to multiple salivary constituents, including amino acids (particularly histidine and glutamine) and carboxylate anions (notably lactate), resulting in characteristic paramagnetic relaxation effects and alterations in their structure and ^1^H NMR profiles. The reversibility of these effects upon EDTA addition confirms the dynamic equilibrium nature of copper–biomolecule binding.

These results have important implications for understanding copper bioavailability, toxicity, and sensory perception in the context of dietary copper intake and exposure to copper-containing materials. The complexation of Cu(II) by salivary proteins and low-molecular-mass biomolecules likely serves as a useful protective function at physiological concentrations, but may be overwhelmed at elevated exposure levels, a process potentially leading to increased oxidative stress and tissue damage in vivo. The relationship between copper ion speciation and metallic taste perception highlights the importance of chemical form in determining sensory responses to metals, however.

Future research should extend these findings to in vivo studies correlating copper ion speciation patterns with physiological and toxicological outcomes, and should apply similar NMR-based approaches to investigate the speciation of other trace metal ions in saliva and further biological fluids. This work establishes a robust methodological framework for investigating metal ion–biomolecule interactions in complex biological matrices, and contributes to our fundamental understanding of trace metal ion biochemistry in the oral cavity.

## Figures and Tables

**Table 2:** FDR-corrected *p* values obtained from ANOVA of the top 22 ^1^H NMR bucket variables, and their directional sign of change. Biomolecular WMSS sample assignments for the buckets are also provided. Abbreviations: ↑ and ↓ represent increases and decreases in intensity, respectively.

Bucket (ppm)	Assignment	FDR-Corrected *p* Value	Direction of Intensity Change
[3.56 .. 3.60]	Gly-CH_2_	1.00 × 10^−53^	↓
[0.96 .. 1.00]	Leu-CH_3_	4.40 × 10^−46^	↓
[3.40 .. 3.44]	Tau-CH_2_SO_3_^−^	2.98 × 10^−43^	↓
[1.32 .. 1.36]	Lac-CH_3_	1.33 × 10^−38^	↓
[1.92 .. 1.96]	A-CH_3_	1.08 × 10^−33^	↑
[1.48 .. 1.52]	Ala-CH_3_	1.86 × 10^−24^	↓
[1.08 .. 1.12]	Unknown	1.17 × 10^−23^	↑
[3.24 .. 3.28]	Tau-CH_2_NH_3_^+^	1.24 × 10^−20^	↓
[0.92 .. 0.96]	n-But-CH_3_	1.26 × 10^−20^	↓
[4.12 .. 4.16]	Lac-CH	8.83 × 10^−17^	↓
[6.88 .. 6.92]	Tyr-C3H/C5H	3.18 × 10^−16^	↓
[1.04 .. 1.08]	Val-CH_3_	4.89 × 10^−15^	↑
[2.16 .. 2.20]	Prop-CH_2_/Gln-β-CH_2_	5.13 × 10^−15^	↑
[3.04 .. 3.08]	Cr-N(CH_3_)/Lys-ε-CH_2_	2.68 × 10^−14^	↓
[2.88 .. 2.92]	TMA-(CH_3_)_3_	7.86 × 10^−14^	↑
[3.28 .. 3.32]	TMAO-(CH_3_)_3_	1.10 × 10^−13^	↑
[4.08 .. 4.12]	Cr-CH_2_	2.39 × 10^−12^	↓
[1.36 .. 1.40]	Acetoin-CH_3_	4.11 × 10^−12^	↓
[1.12 .. 1.16]	*iso*-Prop-CH_3_	6.56 × 10^−12^	↓
[2.20 .. 2.24]	Acetone-CH_3_	1.04 × 10^−10^	↓
[3.20 .. 3.24]	Choline-N(CH_3_)	3.50 × 10^−10^	↑
[1.44 .. 1.48]	Ile-γ-CH	1.52 × 10^−09^	↓

**Table 3:** Statistical significance of the ‘between-added Cu(II) ion’ concentrations (A), ‘between-participants’ (B) and the first-order A × B interaction effect for selected relatively intense variable buckets putatively affected by downfield shifts in their intensities. Abbreviation: ns, not significant.

Metabolite (Coupling Pattern/^1^H NMR Bucket)	*p* Value Between-Added Cu(II) Levels Effect (A)	*p* Value Between-Participants Effect (B)	*p* Value A × B Interaction
Acetate-CH_3_ (*s*/1.92–1.96 ppm)	8.36 × 10^−7^	ns	ns
Alanine-CH_3_ (*d*/1.48–1.52 ppm)	2.59 × 10^−16^	1.26 × 10^−6^	2.10 × 10^−2^
Lactate-CH_3_ (*d*/1.32–1.36 ppm)	4.89 × 10^−10^	7.30 × 10^−3^	ns
N-Acetylsugar-NHCOCH_3_ (*s*/2.04–2.08 ppm)	2.00 × 10^−2^	ns	ns
Propionate-CH_3_ (*t*/1.04–1.08 ppm)	4.66 × 10^−6^	<10^−20^	ns
Succinate-CH_2_s (*s*/2.40–2.44 ppm)	2.00 × 10^−2^	ns	ns

**Table 4:** Correlation matrix for the elemental compositions of solid precipitates/deposits generated from the addition of increasing levels of Cu(II) ion to WMSS samples. Elements were determined by SEM/EDS analysis, and all samples were thoroughly washed with ice-cold distilled water and dried prior to analysis.

Element	Cu	C	O	P	S	N
Cu	1	−0.604	0.155	0.128	0.291	−0.577
C	−0.604	1	−0.622	−0.068	−0.354	0.836
O	0.155	−0.622	1	−0.463	−0.384	−0.480
P	0.128	−0.068	−0.463	1	0.823	−0.248
S	0.291	−0.354	−0.384	0.823	1	−0.395
N	−0.577	0.836	−0.480	−0.248	−0.395	1

**Table 5:** Tabulated list of maximum absorption frequencies of bands present in FTIR-ATR spectral profiles acquired on thin films of control WMSS samples (coded 1–9), alongside those detectable in corresponding spectra of dried Cu(II) deposits formed (coded A–N). Abbreviations: *sh*, shoulder; *v*, *vas*, and *vs* refer to stretching, asymmetric and symmetric vibrations; δ, deformation vibration; ρb, bending vibration; SIgA, immunoglobulin A.

Band Code	Absorption Band Frequency (cm^−1^)	Biomolecular Assignment(s)
1	527	*v*(OCN) (tentative)
A	558	Cu(II)-O stretch (tentative)
B	969	*vs*(C-N-C) Proteins/α-Amylase (tentative)/DNA Backbone (tentative) *ν*(PO_2_^−^) Inorganic Phosphates
2	990	*vs*(C-N-C) Proteins/α-Amylase (tentative)/DNA Backbone (tentative) *ν*(PO_2_^−^) Inorganic Phosphates
C	1019 (sh)	Glycogen (C-O stretch)/Polysaccharides
D	1043	α-Amylase/Glucose, Glycogen and Sugar Residues (C-C and C-O stretches) *ν*(CO), *ρb*(C–O–H) Glycosylated α-amylase, Mucins or Alternative Sugar Residues
3	1074	*vas*(C-N-C) Sugar Residues, Glycosylated Proteins, Proteins and Phosphate Compounds/Phospholipids *vs*(C-O-C stretch)/*vs*(C-O) stretch *νs*(PO_2_^−^) Inorganic Phosphates *νs*(PO_2_^−^), *νas*(PO_2_^−^) Phosphate Group of Phospholipids *ν*(CO), *ρb*(C–O–H) Glycosylated α-amylase, Mucins or Other Sugar Residues
E	1080	*vas*(C-N-C) Sugar Moieties/Glycosylated Proteins/Proteins/Phosphate Compounds/Phospholipids *vs*(C-O-C stretch)/*vs*(C-O) stretch *νs*(PO_2_^−^) Inorganic Phosphates *νs*(PO_2_^−^), *νas*(PO_2_^−^) Phosphate Group of Phospholipids *ν*(CO), *ρb*(C–O–H) Glycosylated α-amylase, Mucins or Other Sugar Residues
4	1240	Amide III/Assymetric and Symmetric Stretch PO_4_/Ester Bond/Phospholipids (*ν*(C–N) of Proteins in α-Helix Conformation)
F	1245 (sh)	Amide III/Assymetric and Symmetric stretch PO_4_/Ester Bond/Phospholipids (*ν*(C–N) of Proteins in α-Helix Conformation) *νas*(PO_2_^−^) Phosphate Group of Phospholipids *νas*(PO_2_^−^) Inorganic Phosphates
G	1381	Proteins *vs*(CH_3_)
5	1408	Proteins *vs*(CH_3_) *νs*(CO_2_^−^) Fatty Acids *ρb*(CH_3_) Proteins
H	1447	δ(N-H) Proteins/*vas*(CH_3_)Lipids
I	1542	Amide II band (N-H bending/C-N and ν(C-C) proteins, tyrosine) tyrosine-containing proteins (α-amylase, albumin, mucins, proline-rich proteins and SIgA) *ρb*(NH), *ν*(C=N), *ν*(C=C)
6	1554	Amide II band (N-H bending/C-N and ν(C-C) proteins, tyrosine) Tyrosine-containing proteins (α-amylase, albumin, mucins, proline-rich proteins and SIgA) *ρb*(NH), *ν*(C=N), *ν*(C=C)
J	1631	Amide I band (C=O stretching/C=O of carboxylates/carboxylic acids or esters/α-helix/C=N stretching) *ν*(C=O) Amide I: unordered structure *ν*(C=O)/*ν*(C=C) Amide I: β-sheet structure ν(C=C) Amide I: β-sheet structure ν(C=O), δ(CN), δ(NH) Amide I: α-helix
U	1634	Residual Water (H-O-H bending)
7	1636	Amide I band (C=O stretching/C=O of carboxylates/carboxylic acids or esters/α-helix/C=N stretching) *ν*(C=O) Amide I: unordered structure *ν*(C=O)/*ν*(C=C) Amide I: β-sheet structure ν(C=C) Amide I: β-sheet structure ν(C=O), δ(CN), δ(NH) Amide I: α-helix
8	2062	SCN^−^ (-C≡N stretch)
K	2865 (sh)	*νs*(CH_3_) Lipids/Protein Side-Chains *v*(C-H)/NH_4_^+^ *νs*(CH_2_) Lipids (cholesterol and mono-/diacylglycerols)
L	2971 (sh)	*vas*(CH_2_) Lipids/Fatty Acids
M	2981 (sh)	*vas*(CH_2_) Lipids/Fatty Acids/Protein Side-Chains
N	3263	Amide A *vas*(N-H): Nucleic Acids/Primary and Secondary Amines
U	3290	Residual Water (O-H stretching)
9	3363 (sh)	Amide A *vas*(N-H): Nucleic Acids/Primary and Secondary Amines

## Data Availability

The data presented in this study are available on reasonable request from the corresponding author.

## Supplementary Materials

The following supporting information can be downloaded at: https://www.mdpi.com/article/10.3390/molecules31152686/s1, Figure S1.1: AHC of the ^1^H NMR-based WMSS Cu(II)-titration dataset using no row-wise normalisation strategy prior to analysis; Figure S1.2: AHC of the ^1^H NMR-based WMSS Cu(II)-titration dataset using a median row-wise normalisation approach prior to analysis; Figure S2.1: plot of mean ± 95% confidence intervals of mean line-width at half-height (Δ*v*_1/2_) versus added Cu(II) concentration for WMSS samples collected from n = 12 participants.
