# Supplementary material for: Investigations of the Molecular Nature of Low-Molecular-Mass Copper(II) Ion Complexes in Human Saliva: Physiological and Toxicological Significance
Source: Molecules. 2026 Aug 1;31(15):2686. doi: 10.3390/molecules31152686 (PMC13468485; doi:10.3390/molecules31152686)
Supplement: Supplementary file 1 [file molecules-31-02686-s001.zip › molecules-4386796-Figure S1.1 and S1.2.pdf]

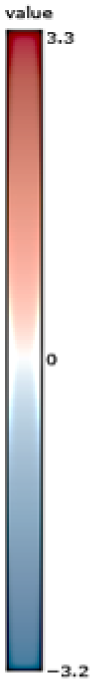
value
3.3
0
−3.2

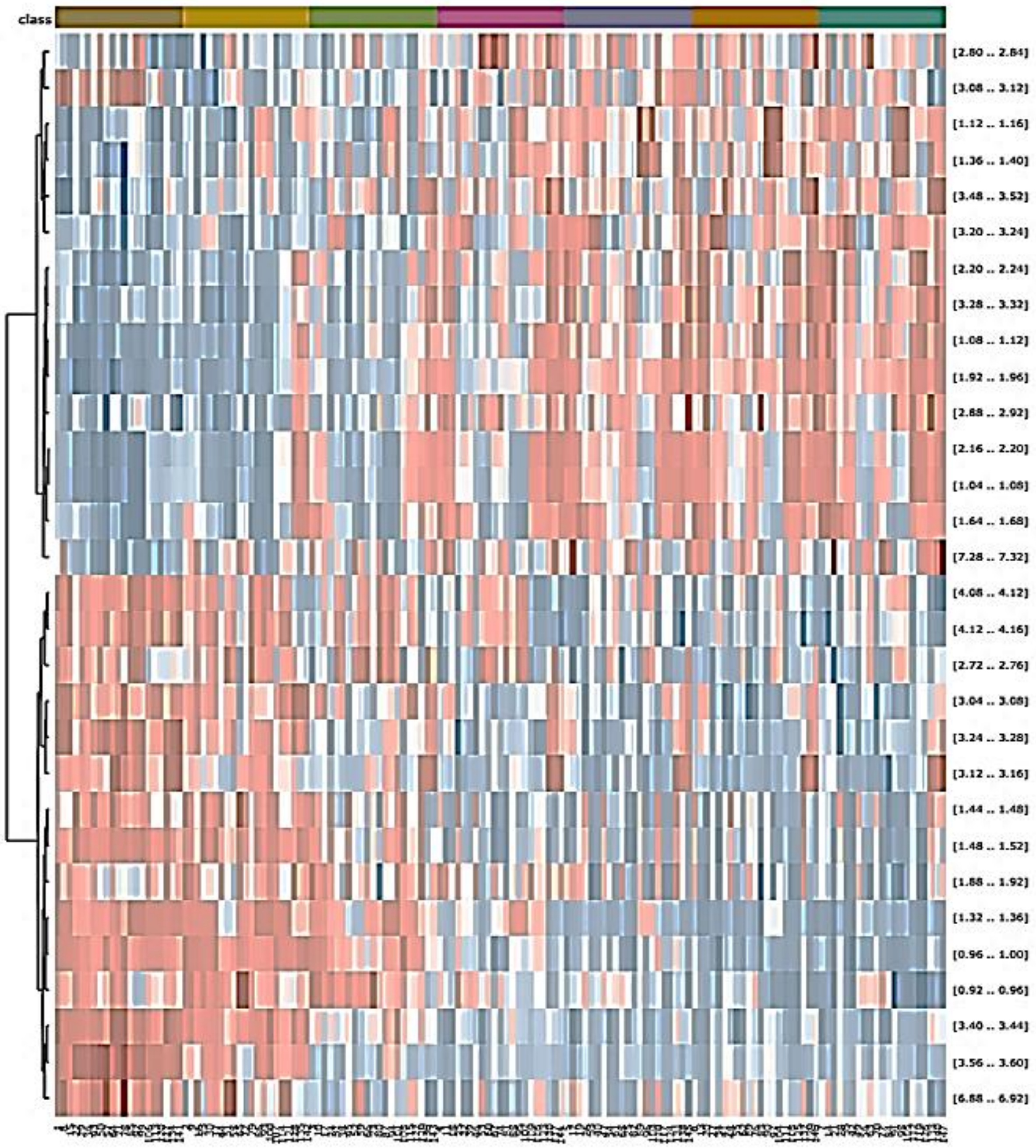

NO ROW-WISE SCALING

Figure S1.1: AHC clustering of the ^{1}H NMR-based WMSS Cu(II)-titration dataset using no row-wise normalisation strategy prior to analysis.

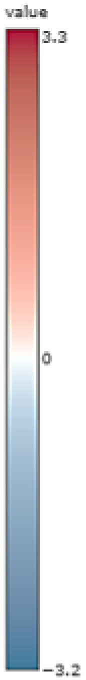
value
3.3
0
−3.2

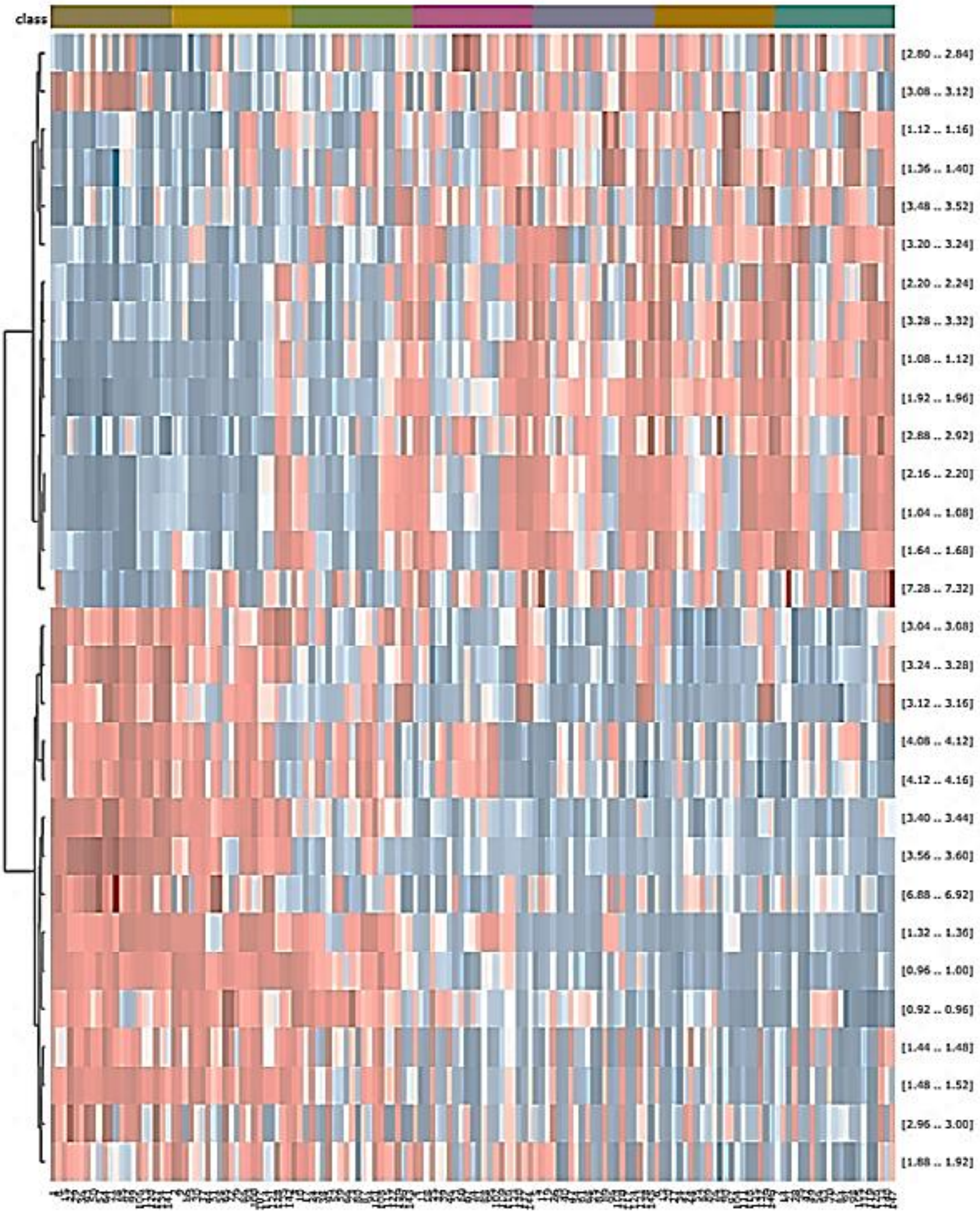

MEDIAN ROW-WISE SCALING ONLY

Figure S1.2: AHC clustering of the ^{1}H NMR-based WMSS Cu(II)-titration dataset using a median row-wise normalisation approach prior to analysis.
