# Supplementary material for: Investigations of the Molecular Nature of Low-Molecular-Mass Copper(II) Ion Complexes in Human Saliva: Physiological and Toxicological Significance
Source: Molecules. 2026 Aug 1;31(15):2686. doi: 10.3390/molecules31152686 (PMC13468485; doi:10.3390/molecules31152686)
Supplement: Supplementary file 1 [file molecules-31-02686-s001.zip › molecules-4386796-Figure S2.1.pdf]

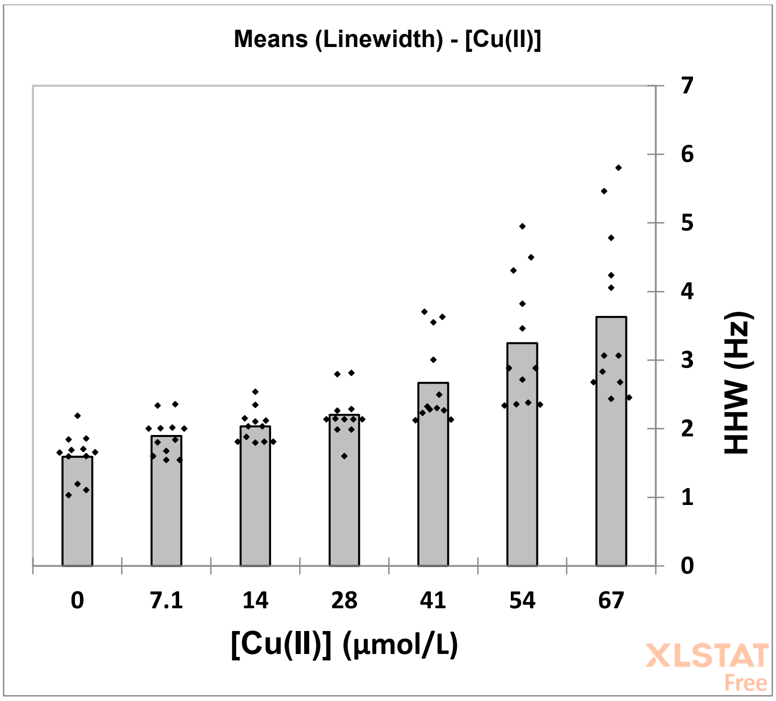

Figure S2.1. Plot of the half-height width of all TSP resonances as a function of added copper(II) ions.
